# Variable selection for clinical prediction models in low-dimensional data - a simulation study comparing traditional regression and machine learning methods

**DOI:** 10.1186/s12874-026-02930-0

**Published:** 2026-07-07

**Authors:** Johannes A. Vey, Georg Heinze, Meinhard Kieser

**Affiliations:** 1 Institute of Medical Biometry, University of Heidelberg, Im Neuenheimer Feld 130.3, Heidelberg, 69120 Germany; 2 Institute of Clinical Biometrics, Center for Medical Data Science, Medical University of Vienna, Spitalgasse 23, Vienna, 1090 Austria

**Keywords:** Clinical prediction models, Variable selection, Regression models, Machine learning, Neutral comparison study

## Abstract

**Purpose:**

A wide range of methods exist for developing a clinical prediction model (CPM) and for performing variable selection. Our purpose was to develop a fair simulation study design and to investigate the properties, strengths, and weaknesses of different methods to predict a continuous outcome in low-dimensional data situations.

**Methods:**

In this simulation study, we conducted a neutral comparison of traditional (linear regression with stepwise selection) and machine learning (regularized regression with elastic net, gradient boosting, random forest) variable selection strategies to derive a CPM. The generated datasets included a total of 15 variables, with 8 of those being predictor variables. Four data- and outcome-generating mechanisms with increasing complexity produced data structures typical for biomedicine covering linear associations and gradually introducing non-linear and non-additive elements into the data structure.

**Results:**

All methods generally performed better with increasing sample size and less noise in the data. Gradient boosting with regression models and with trees as base learners, and the elastic net regularized regression included nearly all variables (i.e., both the predictor and non-predictor variables), especially with increasing sample size. The linear regression model with stepwise selection (LMSS) showed the best trade-off between correctly including the predictors and excluding the non-predictor variables in most of the scenarios, even when the functional form of continuous predictors deviated from linearity. In more complex data, variable selection using the Boruta or Hapfelmeier approach for random forest performed similar to LMSS.

**Conclusion:**

The sample size must be sufficiently large to enable the methods to reliably identify the predictor variables and to ensure that the developed CPMs are accurate and well-calibrated. LMSS revealed good properties and the random forest with the Boruta or Hapfelmeier approach are suitable alternatives if complex associations between predictors and outcomes are assumed.

**Supplementary Information:**

The online version contains supplementary material available at 10.1186/s12874-026-02930-0.

## Background

Clinical prediction models (CPM) aim to provide an individual’s risk or probability of the occurrence of a specific event in the future (prognosis) or the current presence of a particular disease or condition (diagnosis) [1]. CPMs can be utilized to support decisions that have to be made daily by healthcare providers and are increasingly finding their way into clinical research and healthcare. In the current era of evidence-based medicine, clinical decisions are not based solely on subjective opinions but on the most objective evidence. Data-driven support by CPMs can be considered part of the external evidence body and should be adequately integrated since medical decisions are pivotal for the patients’ condition and cure. An example of a prognostic CPM is QRISK^®^3 [2] that provides the 10-year risk of cardiovascular disease, e.g., stroke or heart attack. The QRISK^®^3 model estimates the probability of developing a cardiovascular disease over the next ten years based on risk factors like age, sex, diabetes status, cholesterol level, systolic blood pressure, etc. The estimated individual’s risk can be used to support decisions about preventive measures or accentuate medical advice about the lifestyle. For diagnostic purposes, a CPM can be used to estimate the probability that a specific condition is present. It often uses non-invasive and easy-to-acquire information and can be utilized for early detection of, e.g., cancer at lower expense and with a lower burden for the patient than, e.g., biopsy, magnetic resonance imaging, or computed tomography scan.

A major concern in developing a CPM is that one is often confronted with many candidate predictors available. The fewer parameters needed, the greater the ease of use of the CPM and the less effort required for data collection and application of the CPM in clinical routine. Furthermore, the wrong inclusion of irrelevant noise variables and incorrect exclusion of relevant predictors can seriously affect the accuracy and reliability of the CPM. Including noise variables can lead to overfitting and poorer generalization when evaluated on new data [3, 4]. Even though overfitting is a major issue with high-dimensional data, it also matters with low-dimensional data because, in general, the risk of overfitting increases when the number of candidate predictors increases or the given sample size decreases [5]. Even though variable selection might raise the variance of the predictions, the identification of predictor variables is a crucial aspect of developing a CPM. Indeed, it is important not only to select the relevant variables, but also the choice of the model plays a central role. On the one hand, conventional linear and generalized linear regression models have proven their value in biostatistics. They can also be used as CPMs in combination with variable selection strategies like stepwise selection. Regularized regression methods like lasso [6] or elastic net [7] conduct variable selection and address overfitting by shrinking the estimated predictor effects toward the null. On the other hand, machine learning (ML) algorithms such as random forest [8] or gradient boosting [9–11] with intrinsic variable selection are alternatives to cope with these tasks. Both approaches have their strengths and weaknesses. Regression-based models are well-understood and interpretable, but they demand certain assumptions and requirements about the data, such as a linear relation between the predictors and the outcome. In real data situations, regression models often require adaptions or additional steps, e.g., transformations of the variables and the outcome variable to attain linear relations or imputation of missing values. Without conducting the appropriate adjustments or methods, the regression model will likely suffer from misspecification. ML models are designed to manage complex, e.g., non-linear or non-additive relations, and can handle many candidate variables. Hence, the model architecture and the functional forms are inferred directly from the data, reducing the need for decisions to be made by the user.

To develop a CPM, which ideally includes relevant predictors and excludes irrelevant variables, based on a low-dimensional dataset, a variety of suitable methods is available. This raises the question of which method to choose. In order to give guidance to empirical researchers about the method’s strengths and weaknesses, we endeavored to conduct a neutral comparison study [12] of different traditional (linear regression with stepwise selection) and modern ML variable selection strategies (regularized regression with elastic net, random forest, gradient boosting) with the aim to develop a CPM for a continuous outcome. To enable this, our first objective was to develop a fair simulation design including typical biomedical data structures. The simulated datasets contained a total of 15 variables, which included eight predictor variables and seven variables that, conditional on the predictors, were unrelated with the outcome. We aimed at investigating the methods’ properties, strengths, and weaknesses in varying complexity when using default implementations in R [13]. Another objective was to compare the performance when the methods were forced to supply sparse models including a maximum of four variables. To our knowledge, no such comparison has been conducted up to now.

The article is structured as follows: First, the applied methods are introduced and the design of the simulation study is described. Next, the simulation study results are presented with respect to the variable selection properties and the predictive performance. We summarize the findings in suggesting preferable methods for particular data situations. Further, we apply the methods to a real dataset to predict the intraoperative blood loss for patients who underwent liver transplantation. We conclude by discussing our findings.

## Methods

In this section, we briefly describe the general methods applied in the comparison.

### Linear regression

Backward elimination (BE), forward selection (FS), and stepwise selection, which combines both BE and FS, are popular techniques for selecting variables based on a linear (generalized) regression model. Information criteria that penalize the model fit for model complexity, like the Akaike Information Criteria (AIC) or Bayesian Information Criteria (BIC), or a nominal significance level can be used as selection criteria in each step [14].

### Regularized regression

Regularization techniques for regression modeling include ridge regression [15], the lasso [6], and the elastic net [7]. Ridge regression applies an $L_2$ penalty to the regression coefficients, adding the squared values of the coefficients to the loss function. Lasso regression uses an $L_1$ penalty, incorporating the absolute values of the coefficients, and the elastic net combines the $L_1$ and $L_2$ penalties. Its mixing parameter $\alpha$ controls the balance between the $L_1$ and $L_2$ regularization in the penalty term of the elastic net, and for all three techniques the parameter $\lambda$ controls the overall strength of the penalty. These two hyperparameters are typically determined by k-fold cross-validation (CV) [16].

### Multivariable fractional polynomials

The multivariable fractional polynomials (MFP) algorithm combines variable selection via backward elimination and selection of functional forms for continuous variables. For each continuous variable $x$, the functional form can be comprised of one fractional polynomial (FP) term ($\beta _{1}x^{p_1}$) or two such terms ($\beta _1 x^{p_1} + \beta _2 x^{p_2}$), where the powers $p_1$ and $p_2$ are chosen from a pragmatically restricted set of Box-Tidwell power transformations [17]: $S$={-2, -1, -0.5, 0, 0.5, 1, 2, 3}, where $x^0$ is defined as $\text {log}(x)$. If $p_1=p_2$, a so-called repeated power FP is defined as $\beta _1x^{p_1} + \beta _2x^{p_1}\cdot \log (x)$. The selection of the FP functions and the elimination of variables can be based on a closed testing procedure with a nominal significance level, the AIC, or the BIC. The algorithm iteratively cycles through all variables until convergence of the chosen set of variables and functional forms [18].

### Gradient boosting

Boosting is a versatile technique with the concept of combining multiple weak learners to build a prediction model. In particular, gradient boosting minimizes a loss function by iteratively adding a new base learner to the previous model. Common choices for base learners are (generalized) linear regression models and decision trees, leading to model-based and tree-based gradient boosting, respectively. At each iteration, the candidate base learners are separately fitted to the negative gradient of the loss function, and the best one is selected to update the model. For this, the effect of the best-fitting base learner is multiplied by a small step-length factor $\nu$, typically $\nu$ = 0.01 or 0.001. The main tuning parameter is the stopping iteration $m_\text {stop}$, which can be optimized by k-fold CV, controls the variable selection properties, and implies shrinkage of the predictor effects [19, 20].

### Random forest

A random forest (RF) consists of an ensemble of classification or regression trees and uses bootstrap aggregation (’bagging’) of predictions [8]. Each tree is grown independently on a subset of observations randomly drawn with replacement. Additionally, only a random subset of variables is considered for splitting in each node of a tree. The main tuning parameter, commonly denoted as *mtry*, controls the number of candidate variables evaluated for splitting. For predicting new data, the predictions from all trees are aggregated. Since variable selection is not intrinsically accomplished, an additional approach is needed. Various procedures exist, of which the method by Hapfelmeier [21] and the Boruta method [22] were recommended based on comprehensive simulation studies [23, 24]. Both methods utilize permutation tests using the variable importance (VIMP) measure to select variables. The Boruta procedure iteratively removes variables by adding so-called shadow copies, which are permuted versions of the variables, and comparing the VIMP of each original variable against the highest VIMP among the permuted copies. In contrast, the method by Hapfelmeier compares the VIMP of a variable directly against the VIMP of its permuted version, and incorporates a sequential permutation test framework allowing for early stopping.

## Simulation study

The setup of our simulation study was specified in a protocol published to OSF before we started the study [25]. The protocol contains detailed elaborations, here we briefly describe the following simulation study within the ADEMP framework (Aims, Data-generation, Estimands, Methods, Performance measures) according to the recommendations by Morris [26]. The simulation study was performed using the statistical programming language R version 4.4.2 [13].

### Aims

We aimed to compare different techniques of traditional (stepwise selection) and ML (elastic net, random forest, gradient boosting) methods in their ability to identify predictor variables in realistic low-dimensional data situations typical for, e.g., observational studies and data structures of different complexity with a continuous outcome. In addition, we aimed to compare the derived CPMs regarding their predictive accuracy and their overfit on new data.

### Data-generating mechanism

The simulation study comprised four settings (A, B, C, D) of gradually increasing complexity regarding the structure of the data and associations to the outcome. Each of the four complexity levels has its own data-generating mechanism (DGM) creating $p=15$ variables and their structure, as well as an outcome-generating mechanism (OGM). An overview is given in Table 1. The OGMs generates the outcome variable based on $J=8$ predictors, but do not contain the $J_{nopred}=7$ non-predictor variables. The general underlying OGM has the form:

1 $$\begin{aligned} y_i&= \beta _0 + \sum \limits _{j\in J_{\text {lin}}}\beta _j x_{ij} + \sum \limits _{j\in J_{\text {nonlin}}}f_j(x_{ij})\nonumber \\&\quad + \sum \limits _{(j,k) \in J_{\text {inter}}} g_{jk}(x_{ij}, x_{ik}) + \epsilon _i \end{aligned}$$

with continuous outcome variable $y_i, i=1,\dots ,n$, an index variable denoting the hypothetical independent subjects, $j=1,\dots ,p$, an index for the predictor variables $x_{ij}$, intercept $\beta _0$, and normally distributed error term $\epsilon _i \sim N(0,\sigma ^2)$. $J = \{J_{\text {lin}}, J_{\text {nonlin}}, J_{\text {inter}}\}$ is defined as the set of predictor variables, and the model contains three additive terms of predictor subsets: (1) The set of predictors $J_{\text {lin}} \subset J$ includes predictors with truly linear effects $\beta _j$, and continuous, ordinal, and nominal scale; (2) The second set $J_{\text {nonlin}} \subset J$ includes continuous predictors with truly non-linear effects of functional form $f_j$, and is disjoint from set $J_{\text {lin}}$; (3) The third set $(j,k) \in J_{\text {inter}}$ comprises the interaction of two variables $j, k \in \{1, \dots , J\} \quad \text {with} \quad j \ne k$ and the functional form $g_{jk}$.

**Table 1:** Correlation structure between the variables and functional forms of the predictors to the outcome variable of the complexity levels A-D

Setting	Correlation	Functional form	Interaction
A	linear, non-linear	linear	-
B	linear, non-linear	linear, non-linear, non-monotone	-
C	linear, non-linear, non-monotone	linear, non-linear, non-monotone	-
D	linear, non-linear, non-monotone	linear, non-linear, non-monotone	one interaction

The true VIMP of each predictor in the OGM is described by its partial coefficient of determination $\Delta R^2$ in Table S1 of Additional file 1. It was calculated based on a simulated dataset of $n=100,\!000$ as the difference between the $R^2$ of the respective OGM with the true predictors *J* compared to the $R^2$ of the model that did not contain predictor *j*, that is $J/\{j\}$:

2 $$\begin{aligned} \Delta R^2 = R_{J}^2 - R_{J/\{j\}}^2 \quad . \end{aligned}$$

The DGM and OGM of setting B are based on the simulation design proposed by Binder et al. 2011 [27], which was created by mimicking the scale, distribution, and correlation structure of the variables of a dataset of the German Breast Cancer Study Group (GBSG) [28]. Furthermore, linear and non-linear relations of some of these associations with the outcome were assumed. For the other complexity levels, the DGM and OGM were simplified or extended, but all generated datasets included a total of $p=15$ variables, with $J=8$ of those being predictor variables and 7 non-predictor variables (3 binary, 3 continuous, 1 categorical with 3 levels). As shown in Table 1, complexity level A entailed the simplest DGM and OGM with only linear functions of the predictors to the outcome. The variables were only monotonically and mostly linearly correlated with each other. Complexity level B had the same underlying correlation structure as A (same DGM), but some continuous predictors had non-linear or non-monotone functional forms in the OGM. In complexity level C, non-monotone correlations between the variables were introduced in the DGM, but the outcome was generated applying the same OGM as in B. The greatest complexity was comprised in setting D by including an interaction effect between two predictor variables in the OGM. The OGMs are specified in equations (1)-(4) of Additional file 1, and the predictors and their functional forms are summarized in Table 2. To construct different data availabilities and signal-to-noise ratios, we varied the sample size of the training data to contain $n \in \{100,\,250,\,500,\,1,\!000\}$ observations and the standard deviation $\sigma ^2$ of $\epsilon _i \sim \mathcal {N}(0, \sigma ^2)$ to achieve explained variation by the OGM of $R^2 \in \{0.3,\,0.5,\,0.8\}$, respectively. The full factorial design resulted in 48 scenarios, and 500 datasets were simulated for each scenario ($Q=500$ repetitions). A test dataset was generated with $n_{\text {new}}=100,\!000$ observations by the respective DGM and OGM to evaluate the predictive performance.

**Table 2:** Type of predictor variables and functional form of the continuous predictors of complexity levels A-D. The functional form of predictor$x_{11}$is non-linear if$x_8=0$and linear if$x_8=1$

Predictor	Type	Functional form of continuous predictors			
		Setting A	Setting B	Setting C	Setting D
$x_1$	continuous	linear	non-monotone	non-monotone	non-monotone
$x_3$	continuous	linear	non-monotone	non-monotone	non-monotone
$x_4$	ordinal (3 levels)	-	-	-	-
$x_5$	continuous	linear	non-linear	non-linear	non-linear
$x_6$	continuous	linear	non-linear	non-linear	non-linear
$x_8$	binary	-	-	-	-
$x_{10}$	continuous	linear	linear	linear	linear
$x_{11}$	continuous	linear	linear	linear	interaction with$x_8$

### Methods

We applied the following methods on each simulated dataset. Exemplary code implementing the methods with all specified parameters is given in the Listings 1-7 of Additional file 1 along with further information on the VIMP measures.

- LMSS: Linear regression with stepwise selection employing the AIC was applied using stats::lm and stats::step with the argument direction = "both". We started with the full model, except for the scenarios with $n=100$, where we started with the null model. The standardized regression coefficients were used to estimate VIMP.
- ENET: Regularized linear regression with the elastic net penalty was applied, whereby the hyperparameters $\lambda$ and $\alpha$ were determined by 5-fold CV using glmnet::cv.glmnet. The standardized regression coefficients were used to estimate VIMP.
- MFP: Multivariable fractional polynomial models employing the AIC for variable and function selection was applied using mfp2::mfp2. The VIMP was quantified as the change in AIC resulting from the exclusion of a variable from the finally developed model.
- GBM: Gradient boosting utilizing regression models as base learners was applied using mboost::glmboost with step length $\nu$ of 0.01. The optimal number of boosting iterations $m_\text {stop}$ was determined by 5-fold CV using mboost::cvrisk. The accumulated reduction in the loss function of the base learners was used as VIMP computed by mboost::varimp.
- GBT: Gradient boosting utilizing regression trees as base learners was applied using gbt3::gbmt with step length $\nu$ of 0.01. The optimal number of boosting iterations $m_\text {stop}$ was determined by 5-fold CV using gbt3::gbmt_performance. The accumulated reduction in the loss function of the base learners was used as VIMP computed by gbt3::relative_influence.
- RFB: Random forest with the Boruta method to perform variable selection was applied using Boruta::Boruta. The unscaled permutation importance of the finally developed RF was used as VIMP.
- RFH: Random forest with the Hapfelmeier method to perform variable selection was applied using rfvimptest::rfvimptest. The unscaled permutation importance of the finally developed RF was used as VIMP.

Because the OGM contained some functional forms that could be identified with the MFP algorithm, leading to a non-neutral comparison, MFP was regarded as a benchmark method but not as a competitor. Since RFB and RFH do not provide a final model ready to deploy like the other methods, we trained a RF based on the selected variables using ranger::ranger and determined the *mtry* hyperparameter using 5-fold CV. We dummy-coded the categorical variable and utilized ordinal coding for the ordinal variable [29] for the regression-based methods (LMSS, ENET, MFP, GBM), leading to 17 independent variables available for selection. If a method failed or did not converge, we used a minimal model of the respective method that included only the most important predictor $x_5$ for the respective repetition. In general, the fallback strategy is recommended by Wuensch et al. 2025 [30] and enables the calculation of performance measures across all simulated data sets. Assuming that $x_5$ is a well-known predictor, constructing a model that includes only established predictors can represent a realistic practical choice when data-driven variable selection breaks down.

In a second series of analyses of the simulated data, we developed size-restricted models for each method, which were allowed to only include the four most important predictor variables according to the estimated VIMP, respectively. If less than four variables were selected, only the selected ones were included in the size-restricted model.

Moreover, we fitted an oracle model for each of the methods, which were trained solely considering the eight predictor variables. The ordinal predictor variable $x_4$ required two degrees of freedom in the regression-based models, leading to nine predictor variables. Further, we fitted a true oracle model (TOM) only containing the predictor variables with their true functional forms.

### Estimands

The estimands in this simulation study were the selection status of each variable, whereby the categorical and ordinal variable was considered selected if any of their dummy variables were included in the model. Further estimands were the contribution of the selected variables to the derived CPM by the VIMP, and the predictive performance by means of the root mean squared prediction error (RMSPE) and the calibration slope on the test data.

### Performance measures

In the protocol, we specified various performance measures to investigate different aspects of this simulation study. Because of limited space, we only introduce the most important performance measures for which we present results. For the following performance measures, the indicator function $I(\cdot )$ returns the value 1 if the expression $(\cdot )$ is true and 0 otherwise. We calculated the variable inclusion frequency (VIF) for each variable over the *Q* repetitions: $\text {VIF}_j=\frac{1}{Q}\sum \nolimits _{q=1}^{Q}I(x_j^q=1)$, where $x_j^q=1$ indicates that variable *j* was selected in repetition *q*. To further evaluate the variable selection properties, we calculated the true inclusion frequency $\text {TIF}=\frac{1}{Q}\sum \nolimits _{q=1}^{Q}\frac{\sum \nolimits _{j=1}^{|J|}I(x_j^q, j \in J)}{|J|}$ with $x_j^q$ denoting selected variable *j* in the *q*-th repetition, the true exclusion frequency $\text {TEF}=\frac{1}{Q}\sum \nolimits _{q=1}^{Q}\frac{\sum \nolimits _{j=1}^{|J_{nopred}|}I(x_j^q, j \in J_{nopred})}{|J_{nopred|}}$ with $x_j^q$ denoting excluded variable *j* in the *q*-th repetition, the mean of these two measures to obtain a performance measure, that gives equal weight to correctly identifying predictors and correctly excluding non-predictors, and the model size by the number of selected variables $\text {SV} = \frac{1}{Q}\sum \nolimits _{q=1}^{Q} \text {SV}_q$. Furthermore, we computed the concordance by Kendall’s $\tau$ between the true importance ranking given by $\Delta R^2$ (Table S1 of Additional file 1) and the ranking estimated by the respective VIMP measure: $\tau = \frac{1}{Q}\sum \nolimits _{q=1}^{Q} \tau _q(\Delta R^2, \text {VIMP}_q)$. To evaluate the predictive performance, we calculated the RMSPE: $\text {RMSPE}=\frac{1}{Q}\sum \nolimits _{q=1}^{Q}\sqrt{\frac{1}{n} \sum \nolimits _{i=1}^{n}(\hat{y_i^q}-y_i^q)^2}$. Moreover, we calculated the median calibration slope across the *Q* repetitions to assess the typical shrinkage of the predictions and the median absolute deviation (MAD) of the log(slope) from the ideal value of 0 (=log(1)) to assess the variability in shrinkage [31].

Furthermore, to facilitate an integrated appraisal of the methods, the results of the simulation study were combined by a composite ranking based on five performance measures: inclusion-exclusion balance which is represented by the mean of TIF and TEF, percentage of selected variables, Kendall’s $\tau$, RMSPE, and calibration slope. To compute this, the results across different $R^2$ scenarios were first merged because the $R^2$ of the data generating model is unknown in practice. For each performance measure, the average value across repetitions was calculated, and the methods were ranked accordingly. The final composite rank for each method was then obtained by averaging its five ranks.

## Results

### Simulation study

Across the 500 simulation repetitions conducted for each of the 48 scenarios, method failure was observed in 70 instances. Notably, all failures occurred at the smallest sample size of 100 observations (see Table S2 in Additional file 1). In these cases, simplified fallback models including only predictor $x_5$ were substituted, and these models completed successfully without further failure. MFP is included in the results as a benchmark method. Therefore, in our assessment we focus on the six competing methods.

#### Variable selection

**Fig. 1:**
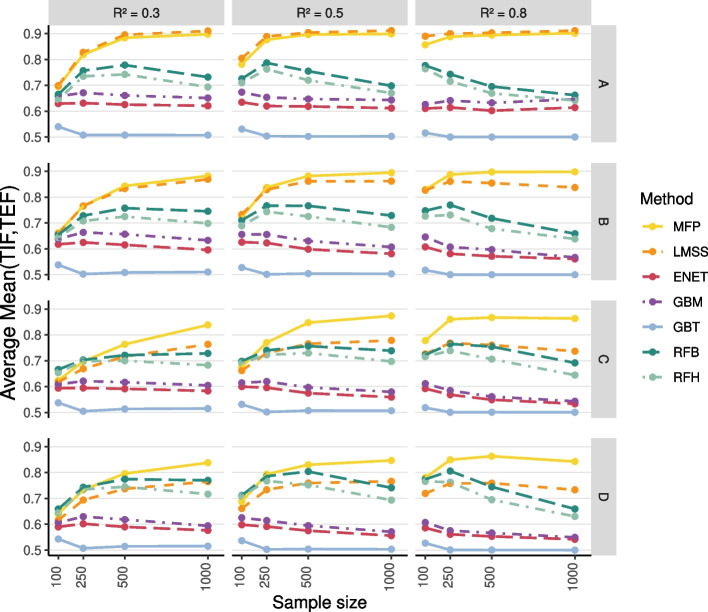
Simulation study: Inclusion-exclusion balance represented by the mean of the true inclusion frequency (TIF) and the true exclusion frequency (TEF) averaged across the 500 repetitions. The plot is stratified by the four complexity levels A-D (row-wise) and the signal-to-noise ratio specified by $R^2$ (column-wise) with the sample size on the x-axis

Figure 1 shows the mean of TIF and TEF as measure for the inclusion-exclusion balance. This measure can assume values between 0 and 1, whereby 1 denotes that all predictor variables are correctly included and all non-predictor variables are correctly excluded. As expected, LMSS outperformed the other methods regarding the selection properties in setting A, where predictors were linearly associated with the outcome. Even in settings B and C, with non-linear effects and correlations, LMSS showed a good compromise between TIF and TEF, comparable to RFB and RFH. In general, the inclusion-exclusion balance of LMSS improved with increasing sample size, except in the scenarios $R^2 = 0.8$. RFB and RFH yielded similar results in the more complex data settings C and D, whereas ENET, GBM, and GBT showed the poorest performance across all scenarios. Naturally, the benchmark method MFP yielded the best such compromise. All methods identified more predictor variables but also more noise variables as the sample size increased (Fig. S5, S6, and S7 of Additional file 1). ENET, GBM, and especially GBT mostly included almost all variables, showcasing a limited ability to perform variable selection in the data situations of our study. The most important predictor variable $x_5$, which had a slightly non-linear effect in B, C, and D, was almost always correctly selected by all methods except when the sample size was 100 (Fig. S8 of Additional file 1). Even the regression-based ones identified $x_5$ well, as its slightly s-shaped effect function could be reasonably approximated by linear functions. It should be noted that the use of the fallback model, which includes $x_5$ whenever a method fails, leads to an increase in the observed VIF of $x_5$; however, this occurs only in the scenarios with n = 100. The second most important predictor $x_3$ had a quadratic effect on the outcome in settings B, C, and D, and additionally a cubic correlation with the non-predictor variable $x_{13}$ in C and D. It was less often identified, especially by LMSS in complexity levels C and D (Fig. S9 of Additional file 1). Since LMSS, ENET, and GBM were not able to model the quadratic effect function of $x_3$, they often selected the non-predictor $x_{13}$ in settings C and D, which was strongly quadratically associated with $x_3$ and captured parts of its effect. MFP selected variable $x_{13}$ less frequently because it probably correctly identified $x_3$ and its functional form. In setting D, the interaction variable $x_{11}$ was selected less frequently by the regression-based methods (Fig. S10 of Additional file 1). In contrast, the weak binary predictor $x_8$, which contributed to the interaction effect, was identified more reliably by RFB and RFH compared to settings A-C (Fig. S11 of Additional file 1), as these methods naturally incorporate interactions during model building.

**Fig. 2:**
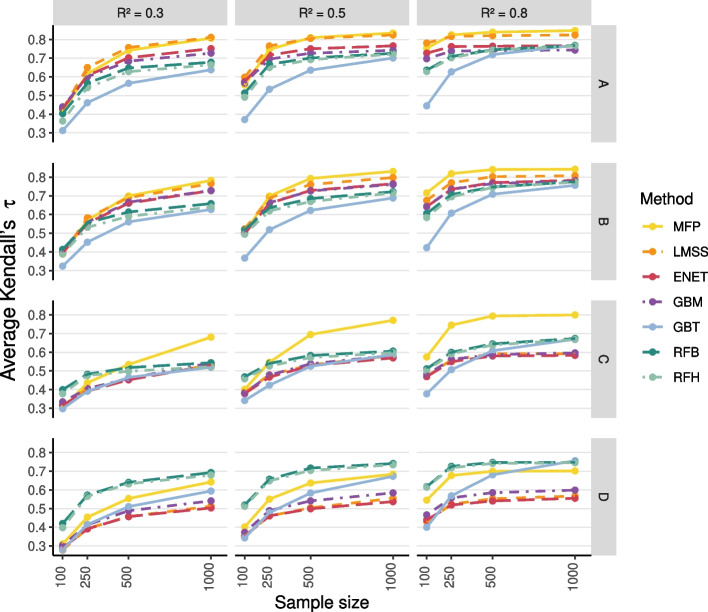
Simulation study: Average of Kendall’s $\tau$ across the 500 repetitions quantifying the concordance of the true ranking of the predictors and the ranking by the variable importance by the methods. The plot is stratified by the four complexity levels A-D (row-wise) and the signal-to-noise ratio specified by $R^2$ (column-wise) with the sample size on the x-axis

To assess the contribution of the variables to the derived CPM, the concordance between the true variable importance ranking by $\Delta R^2$ (Table S1 of Additional file) and the ranking according to the estimated VIMP of the models was estimated by Kendall’s $\tau$. A value of 1 means that the predictor variables were correctly selected, and the ranking through the variable importance exactly matches the true importance of the OGM. Comparing Kendall’s $\tau$ in Fig. 2 shows that the concordance increased with a larger *n*. In the more simple complexity levels A and B, LMSS yielded the best concordance followed by ENET and GBM. The more complex correlation structure in settings C and D generally constitutes a challenge for the regression-based methods as their performance regarding Kendall’s $\tau$ decreased and was exceeded by RFB and RFH.

#### Predictive performance

**Fig. 3:**
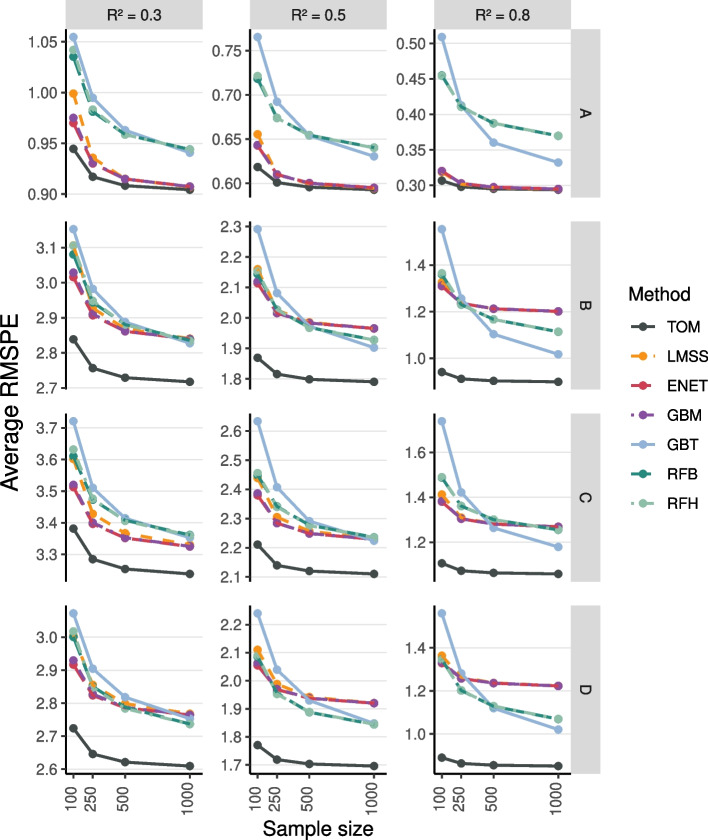
Simulation study: Average RMSPE across the 500 repetitions of the developed models when tested in the test dataset with 100,000 observations. The plot is stratified by the four complexity levels A-D (row-wise), the signal-to-noise ratio specified by $R^2$ (column-wise), and the sample size on the x-axis. Note, because differences between methods are relatively small compared to the large variability across scenarios, each panel uses an independent y-axis scale to better highlight trends within scenarios

Results on the predictive performance in test data in terms of average RMSPE is given in Fig. 3 (see Figure S12 of Additional file 1 for variability). Since the MFP models developed in some repetitions produced very extreme predictions, MFP is not shown in the figures. This behavior is due to the fact that the test dataset was substantially larger than the training datasets, resulting in a wider range of variable values. Consequently, MFP extrapolates beyond the range of the training data, which can lead to extreme predictions. As expected, the true oracle model (TOM) reached the most accurate predictions across all simulated scenarios. Moreover, the predictive performances improved when there was less noise and the variability decreased with increasing *n*. The RMSPE differed little between the methods except in setting A, where there were only linear effects of the predictors on the outcome (complexity level A), and the regression-based methods LMSS, ENET, and GBM showed the lowest prediction error. Furthermore, the regression-based methods, especially ENET and GBM, performed best with small *n* ($n \le 250$) also with the more complex data structures of settings C and D. On the other side, RFB, RFH, and particularly GBT improved the most with increasing *n*. The Monte Carlo standard error of the RMSPE is given in Table S5 of Additional file 1 and was smaller than 0.005 for almost all methods and scenarios. The dependency of the RMSPE on the model size differed between the methods. Considering the scenarios of complexity level C and $R^2=0.5$, providing a realistic data situation, Figure S13 of Additional file 1 revealed that the RMSPE of LMSS reached its minimum with a specific number of selected variables but increased with less or more variables in the model. In contrast, the RMSPE of RFB decreased with the model size, but RFB ensured the exclusion of at least some variables. These trends were evident across all scenarios.

**Fig. 4:**
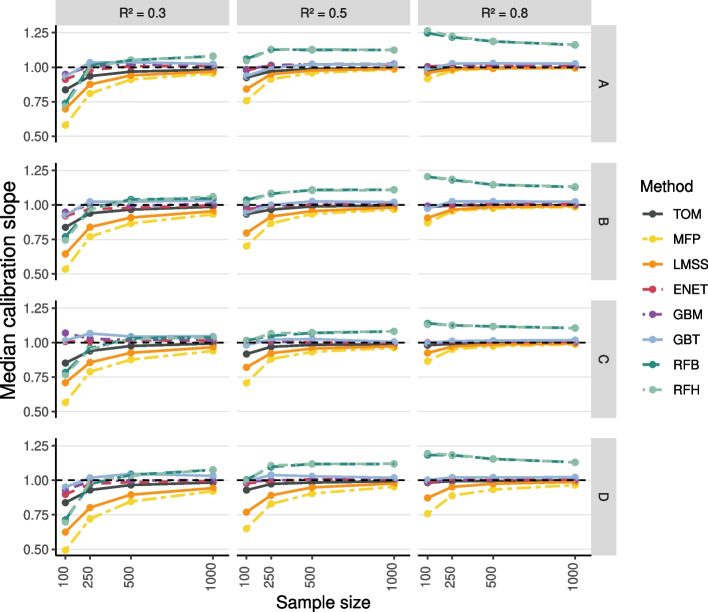
Simulation study: Median calibration slope across the 500 repetitions of the developed models when tested in the test dataset with 100,000 observations. The plot is stratified by the four complexity levels A-D (row-wise), the signal-to-noise ratio specified by $R^2$ (column-wise), and the sample size on the x-axis. The horizontal dashed lines represent the ideal slope of 1

Median calibration slopes are presented in Fig. 4. The calibration slopes approached the target value of 1 as *n* increased, except for RFB and RFH, which showed values greater 1 indicating predictions that were too much shrunken to the mean, particularly in less noisy data. LMSS revealed calibration slopes of less than 1 on median, implying that the predictions are overly extreme. The variability of the calibration slopes decreased with increasing *n* and $R^2$ (Figure S16 and S17 of Additional file 1). The best median calibration was obtained by ENET, GBM, and GBT, because these methods apply shrinkage by design, but GBT revealed a higher variability by the MAD of the log(slope). Considering the calibration slope in dependence of the model size of LMSS and ENET in Figure S18 of Additional file 1, overfitting became more pronounced with an increasing number of included variables, particularly when the sample size was small. Specifically, with 100 and 250 observations, there was a negative correlation between the calibration slope and the number of selected variables. For large models, even ENET exhibited median calibration slopes below 1. With decreasing sample size, the benefit of shrinkage intensified, as shown in the lower RMSPE of ENET compared to LMSS at $n=250$, relative to the difference observed at $n=1000$ (see Figures S14 and S15 of Additional file 1).

The oracle models were trained on datasets solely containing the predictor variables and showed a similar picture of the RMSPE as the developed models (Figure S19 in Additional file 1). The oracle models LMSS-O, ENET-O, and GBM-O showed no remarkable improvement in RMSPE with increasing *n* from $n \ge 250$. RF-O and GBT-O revealed the greatest gain in RMSPE with increasing *n* as observed for their developed models. Figure S20 of Additional file 1 visualizes the difference in RMSPE between the developed models of the methods and their respective oracle model. It shows that the developed models of LMSS, ENET, and GBM had more accurate predictions than their respective oracle model in the scenarios with large *n* and low noise in settings C and D. In these situations, the methods identified almost all predictors but also included additional variables, as seen from the TIF by nearly 100% and decreasing TEF in Figures S5 and S6 of Additional file 1. One of the non-predictors that was frequently selected was $x_{13}$. The continuous variable $x_{13}$ had a reasonable non-linear correlation to the non-linear predictor $x_3$, leading to an approximately linear correlation of $x_{13}$ with the outcome, as shown by the scatter plot in Figure S22 of Additional file 1. The partial residual plots [32] of the linear model show that by missing the true functional form of predictor $x_3$, part of the effect was transmitted to variable $x_{13}$ (Figure S23 of Additional file 1), demonstrating that non-linear effects can be compensated by including correlated variables. If the functional form was correctly captured as in TOM, variable $x_{13}$ had no effect (Figure S24 of Additional file 1). In contrast to the regression-based methods, the developed models of GBT, RFB, and RFH did not reach the RMSPE of their corresponding oracle model, which suggests that RF and GBT benefit from background knowledge.

#### Size-restricted models

Figure S25 of Additional file 1 depicts the TIF of the size-restricted models, which were allowed to include only the four variables with highest importance, respectively. In settings A and B, the size-restricted models performed similarly as their unrestricted counterparts. However, in settings C and D size-restricted LMSS, ENET, and GBM reached only a TIF of around 80%, because they often included the non-predictor variable $x_{13}$ instead of predictor $x_3$. The RMSPE of the size-restricted models were slightly larger than those of unrestricted models, but overall showed a similar pattern (Figure S26 of Additional file 1). The calibration slopes of the size-restricted RFB and RFH also depended on $R^2$, but the median calibration slopes were generally smaller than those of their unrestricted counterparts (Figure S27 of Additional file 1). For $R^2=0.5$, the median calibration slopes of the size-restricted RFB and RFH approached 1, but the predictions in the scenarios with $R^2=0.3$ were too extreme, whereas the predictions were too much shrunken at low noise, except in setting D.

#### Summary measure

In order to combine the results and provide general recommendations on preferable methods for particular data scenarios, we present the best three methods for each combination of sample size and setting in Table 3 with the combined rank given in the brackets. Since six methods were compared, the ranks can range from 1 to 6. The computational time was not considered in the summary measure, because the running time is of minor relevance and depends on the computer’s processing power.

**Table 3:**
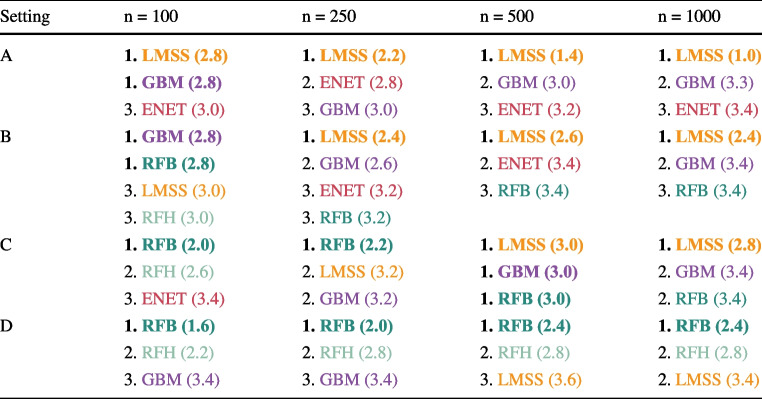
Simulation study: Summary table providing the best three methods according to the mean rank of the ranks of inclusion-exclusion trade-off, number of selected variables, Kendall’s $\tau$, RMSPE, and calibration slope

LMSS was the best-performing method in complexity level A and also ended up in the top three in settings B and C, except in setting C with $n=100$. RFB took the first place in all scenarios in the more complex data settings C and D, except setting C with $n=1,000$, and further, reached a top three rank in setting B.

In terms of computational efficiency, Boruta and particularly the Hapfelmeier approach exhibited the longest running times (Figure S28 of Additional file 1). While these times could be reduced through parallel processing, our study restricted computation to a single core to ensure reproducibility.

### Data example

The methods were applied to a real dataset [33] with the aim to develop a CPM predicting intra-operative blood loss during liver transplantations and to identify the most important predictors. The sample size was 779 and the outcome variable, intra-operative blood loss, was log-transformed to obtain an approximately normally distributed continuous outcome variable. After transformation, the median was 8.01 log(ml), with an interquartile range of 7.39 to 8.61 log(ml). The dataset included 17 variables with continuous (c), ordinal (o), and binary (b) scales, and there were no missing values. The descriptive statistics, distributions, and correlations of all variables are summarized in Table S6 and illustrated in Figure S29 and S30 of Additional file 1.

**Fig. 5:**
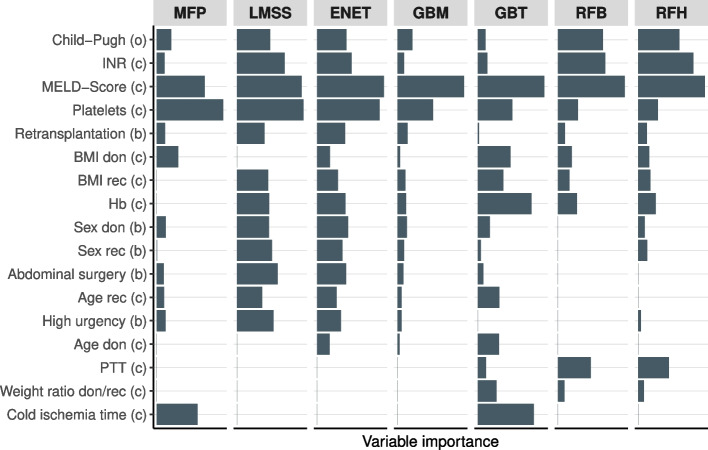
Data example: Selected variables and corresponding variable importance of the methods applied to the real data example predicting intra-operative blood loss

The methods were utilized as in the simulation study and their selected variables are those for which no variable importance is shown in Fig. 5. The Child-Pugh score assessing the prognosis of liver cirrhosis, the international normalized ratio (INR) measuring how long it takes blood to clot, the MELD score indicating the severity of chronic liver diseases, the concentration of platelets, and the binary retransplantation indicator were selected by all methods. Partial thromboplastin time (PTT) and the weight ratio of the donor and recipient were only selected by the tree-based methods. The cold ischemia time was only selected by GBT and MFP, whereby a non-linear association with blood loss was estimated with powers $p_1=0$ and $p_2=0.5$. To evaluate the predictive performance, the developed models were 5-fold cross-validated and the RMSPE and the calibration slope were calculated. Table 4 shows that the RMSPE were similar, but MFP revealed the best RMSPE and acceptable calibration.

**Table 4:** Data example: Model size by number of selected variables, predictive performance by RMSPE, and calibration slope of the methods applied on the real data example predicting intra-operative blood loss

	LMSS	ENET	MFP	GBM	GBT	RFB	RFH
Model size	12	14	12	14	16	10	13
RMSPE	0.831	0.829	0.824	0.832	0.841	0.837	0.833
Slope	0.888	1.05	0.901	0.897	0.909	1.058	1.284

## Discussion

Selecting the most important predictor variables when developing a CPM is a common task in biomedical research, and a wide variety of methods exists. Many investigations focus on high-dimensional data, binary prediction, or compare only similar techniques like traditional regression modeling or machine learning algorithms, respectively. Our investigation aimed to compare traditional and modern strategies for selecting predictor variables to develop a CPM for a continuous outcome based on low-dimensional data. We examined the properties of the applied methods and provide guidance on suitable methods for different scenarios.

ENET, GBM, and GBT conduct variable selection directly during model fitting, which is controlled by the hyperparameters. The hyperparameters are typically determined by resampling strategies minimizing the prediction error, which often results in selecting non-predictor variables [34], which in our study resulted in nearly all variables being included in the final model. The stability selection framework could be applied in combination with these methods to address this concern and enforce the models to be more parsimonious [35, 36]. LMSS revealed competitive properties regarding the identification of predictors, as it was partly able to approximate non-linear functional forms by linear ones. Supposing that true functional forms of predictors strongly deviating from linearity and interactions are known by expert knowledge or uncovered by exploratory data analysis, the linear regression model with stepwise selection may be able to deal with these challenges even better in practice. RF inherently incorporate non-linear correlations and functional forms, as well as interactions when the tree depth exceeds one. Hence, fewer modeling choices concerning the underlying associations are necessary compared with regression-based methods, and RFB and RFH performed variable selection particularly well in the scenarios with complex data of our study.

As stated for binary prediction [37], ML models need larger training data to achieve predictive performance comparable to the linear model for a continuous outcome. The studies of Rothacher [23] and O’Connell [24] investigating various selection methods for RF in data situations similar to ours and recommended the Boruta and Hapfelmeier approach. We were able to show that these approaches were also successful compared to other model techniques. However, in order for these methods to yield superior performance, the sample size should be large enough, based on our findings of around 500-1,000 observations, and there should not be too much noise.

The extreme predictions of MFP for some observations in certain repetitions were caused by extrapolation, occurring when values of a continuous variable in the large test dataset fell outside the range observed in the training data. This situation can lead to an inconvenient combination of the FP function and the shift factor for a continuous variable which was exacerbated by the large test data. For example, the shift factor might be determined that, after transformation, the values of a continuous variable are positive but very close to zero. If the logarithm is then selected, the predicted values get extremely small since $\lim _{x \rightarrow 0} \log (x) = -\infty$. In practical application, this issue can be readily addressed by manually adjusting the shift factor or by truncating the predictor values at the extremes observed in the training data. The real data example demonstrated that MFP has a strong potential to perform competitively.

An important aspect of CPMs is their calibration, where we observed cardinal differences between the methods. As Riley 2019 [38] pointed out, shrinkage decreases as the sample size and $R^2$ increase for linear regression models. This aligns with our findings for LMSS (and TOM) since the calibration slope converged to 1 with increasing *n*. Using equation 9 of Riley 2019 [38] for our scenario of $R^2 = 0.5$ and 17 variables, the calculated minimum sample size would be 191 (408) observations to achieve a calibration slope of 0.9 (0.95). The too moderate predictions of RFB and RFH might be due to the averaging of the individual decision trees and the lack of extrapolation. This behavior applies not only to predicting a continuous outcome but also to binary classification, as Barrenada thoroughly examined [39].

A fair comparison between traditional regression-based and modern ML models with the aid of simulation studies is challenging because the DGM should not favor any of the techniques [40]. We designed a simulation study by adapting and extending the DGM and OGM of Binder et al. [27] providing the basis for a neutral comparison study with realistic data typical for biomedicine. It can be used or modified for further comparison studies, and the R code is available from https://github.com/VeyJo/VarSelCPM. However, our study has some limitations. Firstly, our simulation study was limited to certain scenarios, and, e.g., more variables or a varying proportion of predictor variables would be of interest. Secondly, the complexity levels are not directly comparable because each has its own DGM and OGM, even though we constructed them as similarly as possible. Furthermore, complexity level A is unsuitable for fairly comparing regression models and ML methods because the OGM favors linear models. In addition, its data structure may be too simplistic to reflect real data. Nevertheless, it allowed us to examine the performance difference between regression- and tree-based methods. The regression-based methods were generally disadvantaged in setting D since they were restricted to the main effects and were not allowed to explore interactions between predictors. Thirdly, we assessed the inclusion-exclusion balance by calculating the mean of TIF and TEF using equal weights. However, the weights could be adapted to situations where false exclusions are more problematic than false inclusions, or where inclusion of strong predictors is more relevant than inclusion of weak predictors. Lastly, we used default settings and did not perform excessive hyperparameter tuning except for the most important and necessary hyperparameters. This might lead to models which do not provide optimal performance, but it avoids the higher variability that one would have to expect for excessively tuned models [31, 41, 42]. Despite these limitations, the simulation study allowed us to fairly assess the strengths and weaknesses of default implementations in R, which are widely used.

In the real data application, LMSS, ENET, and GBM selected similar variables, while RFB resulted in the most parsimonious model with good RMSPE and calibration slope. MFP selected the cold ischemia time with a quadratic functional form. Since this variable was not selected by the other regression-based methods, the observed properties in the simulation study might suggest a non-linear effect on blood loss in this dataset.

## Conclusion

Through the developed simulation study design, we compared traditional and ML methods to conduct variable selection when developing a CPM and identified preferable methods in different realistic data situations. LMSS demonstrated good properties even if associations were not linear when non-linear functional forms could be approximated by linear ones or when they could be partly captured by including additional correlated variables. The RF approaches RFB and RFH obtained satisfying results regarding variable selection, parsimony, and prediction. They are particularly recommended in complex data structures or when unknown interactions might exist and when model explainability and transparency is less critical. As commonly known and acknowledged by numerous publications, the sample size must be sufficiently large, with at least 500 observations for the low-dimensional data investigated in our study to obtain satisfactory and stable performances, which confirmed similar conclusions by Riley et al. 2023 [43]. Especially in the context of variable selection, instability should be evaluated in practice using approaches such as the bootstrap [14].

## Supplementary Information

Supplementary Material 1. Accompanying supplementary information are provided in Additional file 1.

## Data Availability

The code for the simulation study, including data generation as well as the application and comparison of methods, is available at https://github.com/VeyJo/VarSelCPM. The clinical data on intra-operative blood loss during liver transplantations cannot be made publicly available due to data protection restrictions.
