# Supplementary material for: Variable selection for clinical prediction models in low-dimensional data - a simulation study comparing traditional regression and machine learning methods
Source: BMC Med Res Methodol. 2026 Jul 7;26:151. doi: 10.1186/s12874-026-02930-0 (PMC13340120; doi:10.1186/s12874-026-02930-0)
Supplement: Supplementary file 1 — Supplementary Material 1. Accompanying supplementary information are provided in Additional file 1. [file 12874_2026_2930_MOESM1_ESM.pdf]

Variable selection for clinical prediction models in low-dimensional data - a simulation study comparing traditional regression and machine learning methods

Supplementary materials

Johannes A. Vey, Georg Heinze, Meinhard Kieser

List of Figures

S1 True functional form of the predictor effects in complexity level A . . . 3
S2 True functional form of the predictor effects in complexity level B . . . 3
S3 True functional form of the predictor effects in complexity level C . . . 4
S4 True functional form of the predictor effects in complexity level D . . . 4
S5 True inclusion frequency . . . 12
S6 True exclusion frequency . . . 12
S7 Model size by percentage of selected variables . . . 13
S8 Variable inclusion frequency of predictor x5 . . . 14
S9 Variable inclusion frequency of predictor x3 . . . 14
S10 Variable inclusion frequency of predictor x11 . . . 15
S11 Variable inclusion frequency of predictor x8 . . . 15
S12 RMSPE of the final models in the test dataset . . . 18
S13 RMSPE in dependence of MS of LMSS and RFB . . . 19
S14 RMSPE in dependence of MS of LMSS and ENET with n=250 . . . 20
S15 RMSPE in dependence of MS of LMSS and ENET with n=1000 . . . 20
S16 Calibration slope of the final models. . . . 21
S17 MAD of the log(slope) of the final models . . . 22
S18 Calibration slope in dependence of model size of LMSS and ENET . . . 23
S19 RMSPE of the oracle models in the test dataset . . . 24
S20 Deviation of RMSPE between the final and oracle models . . . 25
S21 Variable Inclusion Frequency (VIF) across simulation repetitions of non-predictor variable x13. . . . 26
S22 Scatter plot of non-predictor x13 and y . . . 26
S23 Partial residual plots of predictor x3 and non-predictor x13 . . . 27
S24 Partial residual plots of predictor x3 and non-predictor x13 in the true oracle model . 27
S25 True inclusion frequency of the size-restricted models . . . 28
S26 RMSPE of the size-restricted models in the test dataset . . . 29
S27 Calibration slope of the size-restricted models in the test dataset . . . 30
S28 Computational time . . . 31
S29 Distribution of the variables of the real data example. . . . 34
S30 Spearman correlation between the variables of the real data example. . . . 35

List of Tables

S1 Contribution of the true predictor variables to the true model by ΔR^2. 5
S2 Number of runs the methods converged or failed with respect to the sample size. . . . 11
S3 Model size by percentage of selected variables (SV) across all scenarios. 13
S4 Average RMSPE across simulation repetitions of the final models in the large test dataset for all scenarios. 16
S5 Monte Carlo standard error of RMSPE for all scenarios. 17
S6 Description of the outcome and candidate variables of the real data example. 32

Simulation study design

Outcome-generating mechanisms

Since predictor x_4 is an ordinal predictor with three levels (ref, a, b), the notation x_{4a} in the following equations refers to the effect of level a.

Setting A

$$y_i = -0.02x_{i1} + 0.7\left(\frac{x_{i3}+10}{25}\right) - 0.3x_{i4a} - 0.12x_{i5} + 0.0015x_{i6} + 0.2x_{i8} + 0.015x_{i10} + 0.02x_{i11} + \epsilon_i \tag{1}$$

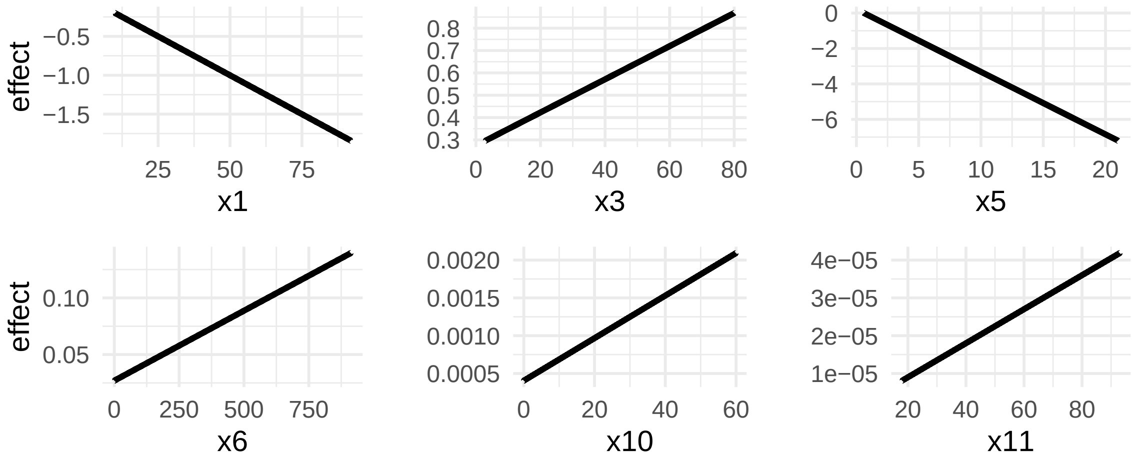

Figure S1: True functional form of the effects of the continuous predictor variables in complexity level A.

Setting B

$$y_i = 3.5x_{i1}^{0.5} - 0.28x_{i1} + 5\cdot\left(log\left(\frac{x_{i3}+10}{27}\right)\right)^2 - 0.8x_{i4a} - 0.37x_{i5} - 1.8\cdot\exp\left(-\frac{(\log(x_{i5})-1.5)^2}{0.4}\right) + 0.42\cdot\log(x_{i6}+1) + 0.55x_{i8} + 0.04x_{i10} + 0.07x_{i11} + \epsilon_i \tag{2}$$

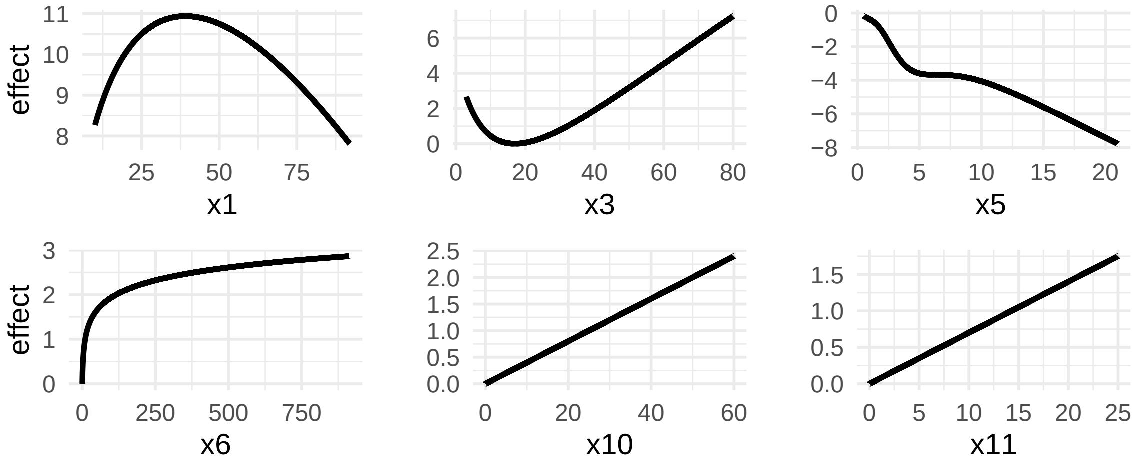

Figure S2: True functional form of the effects of the continuous predictor variables in complexity level B.

Setting C

$$
\begin{aligned}
y_i = {} & 3.5x_{i1}^{0.5} - 0.28x_{i1} + 5 \cdot \left(log\Big(\frac{x_{i3}+10}{27}\Big)\right)^2 - 0.8x_{i4a} \\
& - 0.37x_{i5} - 1.8 \cdot \exp\left(- \frac{(\log(x_{i5}) - 1.5)^2}{0.4} \right) + 0.42 \cdot \log(x_{i6}+1) \\
& + 0.55x_{i8} + 0.04x_{i10} + 0.07x_{i11} + \epsilon_i
\end{aligned} \tag{3}
$$

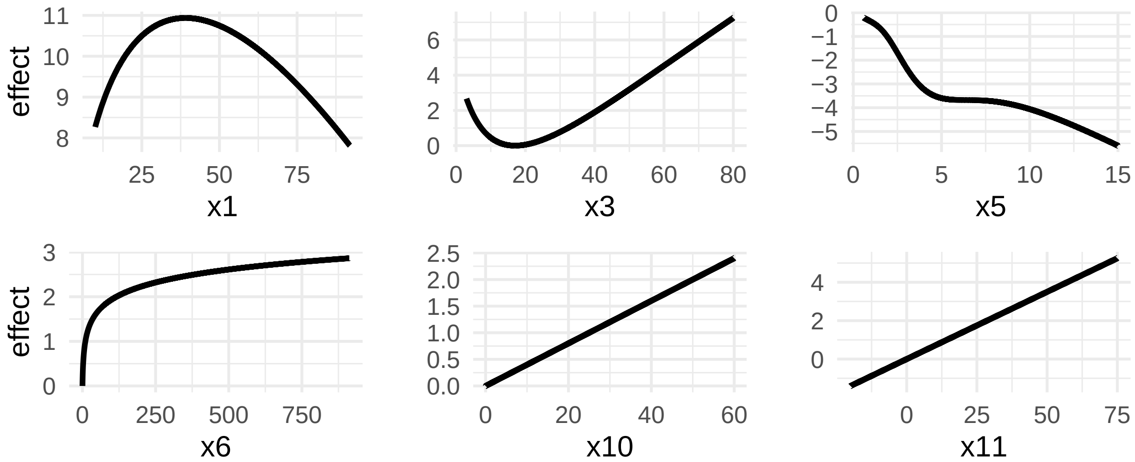

Figure S3: True functional form of the effects of the continuous predictor variables in complexity level C.

Setting D

$$
\begin{aligned}
y_i = {} & 3.5x_{i1}^{0.5} - 0.28x_{i1} + 5 \cdot \left(log\Big(\frac{x_{i3}+10}{27}\Big)\right)^2 - 0.8x_{i4a} \\
& - 0.37x_{i5} - 1.8 \cdot \exp\left(- \frac{(\log(x_{i5}) - 1.5)^2}{0.4} \right) + 0.42 \cdot \log(x_{i6}+1) \\
& + 3x_{i8} + 0.04x_{i10} + \begin{cases} 0.92 \cdot log(x_{i11}+1) & \text{if } x_8 = 0 \\ 1 - 0.04x_{i11} & \text{if } x_8 = 1 \end{cases} + \epsilon_i
\end{aligned} \tag{4}
$$

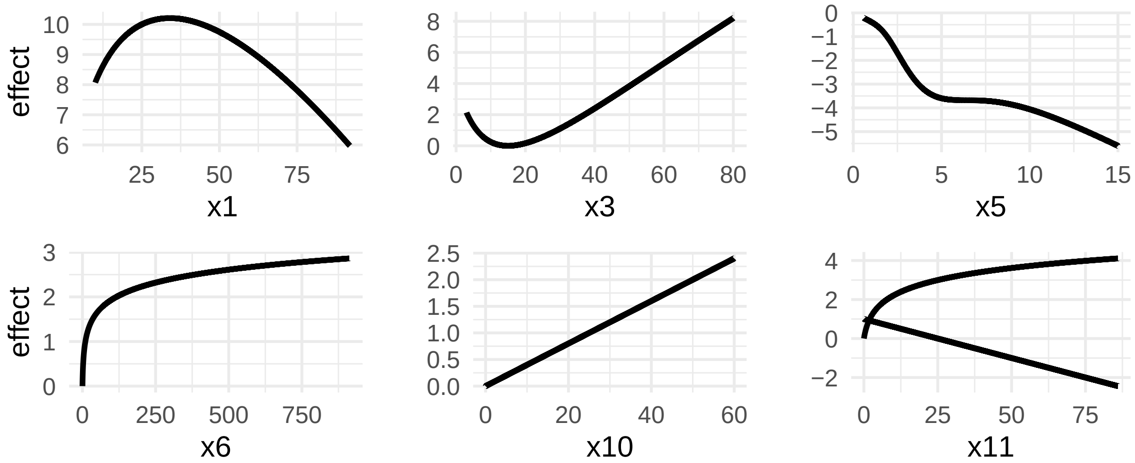

Figure S4: True functional form of the effects of the continuous predictor variables in complexity level D.

Table S1: Contribution of the true predictor variables to the true model by ΔR^2.

	Setting			
**Predictor**	**A**	**B**	**C**	**D**
x1	0.058	0.030	0.023	0.034
x3	0.083	0.069	0.049	0.077
x4a	0.029	0.022	0.016	0.025
x5	0.121	0.134	0.098	0.152
x6	0.054	0.043	0.016	0.062
x8	0.013	0.010	0.008	0.027
x10	0.020	0.016	0.011	0.018
x11	0.044	0.053	0.033	0.046

Methods

Listing 1 Example code to apply MFP

```
# perform variable and FP selection
fit_mfp <- mfp2(y ~ ., data = data,
                center = TRUE, criterion = "aic", verbose = FALSE)

# corresponds to the formula
y ~ fp(x1) + x2 + fp(x3) + x4_1 + x4_2 + fp(x5) + fp(x6) +
    fp(x7) + x8 + x91 + x92 + fp(x10) + fp(x11) + fp(x12) +
    fp(x13) + x14 + x15

# predict test data
predict(fit_mfp, newdata = data_new, type = "response")
```

The variable importance (VIMP) of the MFP model was quantified as the change in AIC resulting from the exclusion of a variable from the finally developed model. This was calculated applying the `stats::AIC` function on the final model (`fit_mfp` here) and the model without the respective variable.

Listing 2 Example code to apply LMSS

```
# if EPV < 10 stepwise forward
fit_lmss <- step(lm(y ~ 1, data = dat),
                 scope = formula(lm(y ~ ., data = dat)),
                 direction = "both")

# if EPV >= 10 stepwise backward
fit_lmss <- step(lm(y ~ ., data = dat),
                 direction = "both")

# predict test data
predict(fit_lmss, newdata = dat_test, type = "response")
```

The standardized regression coefficients were used to estimate VIMP of the final LMSS model. To estimate the VIMP, all variables in the dataset were standardized using the `scale` function, the final linear regression model with the selected variables was fitted, and the resulting regression coefficients were extracted.

Listing 3 Example code to apply ENET

```
# applying 5-fold CV to each value of alpha sequence (0.1-0.9, by 0.1)
seq_alpha <- seq(from = 0.1, to = 0.9, by = 0.1)
list_cv <- list()
for(i in 1:9){
  list_cv[[i]] <- glmnet::cv.glmnet(X, Y, alpha = seq_alpha[i],
                                    nfolds = 5, type.measure = "mse")
}

# extract lowest CV mse of respective alpha/lambda combination
cvm_min <- unlist(lapply(list_cv, function(x)
  mse.min <- x$cvm[x$lambda == x$lambda.min]))

# identify model with lowest CV mse
k <- which.min(cvm_min)

# extract final model
fit_enet <- list_cv[[k]]

predict(fit_enet, s = "lambda.min",
        newx = dat_test, type = "response")
```

The standardized regression coefficients were used to estimate VIMP of the final ENET model. Since the `glmnet` function internally standardizes the variables but returns regression coefficients on the original scale, standardized coefficients were obtained by dividing each returned coefficient by the standard deviation of the corresponding variable.

Listing 4 Example code to apply GBM

```
# train GBM
fit_gbm <- mboost::glmboost(y ~ ., data = dat,
                            family = Gaussian(),
                            control = boost_control(mstop = 10000,
                                                    nu = 0.01,
                                                    risk = "inbag"))

# perform 5-fold CV to estimate best stoping iteration
cv <- mboost::cvrisk(fit_gbm,
                     folds = mboost::cv(model.weights(glmboost),
                                        type = "kfold", B = 5),
                     papply = lapply)

# predictions on new data using final model at m_stop
predict(fit_gbm[mstop(cv)], newdata = dat_test, type = "response")
```

The accumulated reduction in the loss function across the iterations in which the base learner of the corresponding variable was selected was used as VIMP for the final GBM model. This was obtained by the function `mboost::varimp(fit_gbm[mboost::mstop(cv)])`.

Listing 5 Example code to apply GBT

```
# perform 5-fold CV to train gbm
fit_gbt <- gbm3::gbmt(y ~ ., data = dat,
                      distribution = gbm_dist("Gaussian"),
                      train_params = training_params(num_trees = 10000,
                                                     interaction_depth = 3,
                                                     shrinkage = 0.01,
                                                     bag_fraction = 0.5,
                                                     num_train = round(0.5*nrow(dat)),
                                                     num_features = ncol(dat) - 1),
                      cv_folds = 5,
                      par_details = gbmParallel(num_threads = 1))

# estimate best stoping iteration using cv error
mstop <- gbm3::gbmt_performance(fit_gbt, method = "cv")

# predictions on new data using final model at m_stop
predict(fit_gbt, n.trees = best_iter, newdata = dat_test, type = "response")
```

The accumulated reduction in the loss function across the iterations in which the base learner of the corresponding variable was selected was used as VIMP for the final GBT model. This was obtained by the function `summary(fit_gbt, num_trees = mstop)`.

Listing 6 Example code to apply RFB

```
# apply Boruta variable selection
boruta <- Boruta(x = dat[,-1], y = dat[,1],
                 pValue = 0.01, mcAdj = TRUE,
                 maxRuns = 1000, doTrace = 0,
                 holdHistory = TRUE, getImp = getImpRfZ,
                 ntree = 1000,
                 mtry = ceiling(ncol(dat[,-1])/3),
                 num.threads = 1)

# variables will be considered as selected if "Confirmed"
boruta[["finalDecision"]]

# determine best mtry for final model
mtry_seq <- 1:length(<selected variables>)
list_rf <- lapply(mtry_seq, function(m) {
      ranger::ranger(y ~ <selected variables>, data = dat,
                     num.trees = 1000, mtry = m,
                     importance = "permutation", num.threads = 1)
    })

# extract and identify mtry with lowest OOB error
oob_error <- unlist(lapply(list_rf, function(x) x$prediction.error))
k <- which.min(oob_error)

# take final random forest (RFB)
fit_rfb <- list_rf[[k]]

# predictions on new data using RF
predict(fit_rfb, newdata = dat_test, type = "response")
```

The unscaled permutation importance of the final RFB was used as VIMP, which can directly be extracted by `fit_rfb$variable.importance`. Note, the `Boruta` function uses the scaled permutation importance by default.

Listing 7 Example code to apply RFH

```
# conduct variable selection based sequential permutation tests
rf_imptest <- rfvimptest::rfvimptest(data = dat, yname = "y",
                                     condinf = FALSE, type = "SPRT",
                                     ntree = 1000,
                                     mtry = ceiling(ncol(dat[,-1])/3),
                                     num.threads = 1)

# variables will be considered as selected if "accept H1"
rf_imptest$testres

# determine best mtry for final model
mtry_seq <- 1:length(<selected variables>)
    list_rf <- lapply(mtry_seq, function(m) {
      ranger::ranger(y ~ <selected variables>, data = dat,
                     num.trees = 1000, mtry = m,
                     importance = "permutation", num.threads = 1)
    })

# extract and identify mtry with lowest OOB error
oob_error <- unlist(lapply(list_rf, function(x) x$prediction.error))
k <- which.min(oob_error)

# fit final random forest (RFH)
fit_rfh <- list_rf[[k]]

# predictions on new data using RF
predict(fit_rfh, newdata = dat_test, type = "response")
```

The unscaled permutation importance of the final RFB was used as VIMP, which can directly be extracted by `fit_rfh$variable.importance`.

Detailed results of the simulation study

Table S2 shows the number of how often the methods threw an error which was considered as non-converged.

Table S2: Number of runs the methods converged or failed with respect to the sample size.

n	method	convergence	non-convergence
100	MFP	5994	6
100	LMSS	6000	0
100	ENET	5987	13
100	GBM	5973	27
100	GBT	6000	0
100	RFB	5991	9
100	RFH	5985	15
250	MFP	6000	0
250	LMSS	6000	0
250	ENET	6000	0
250	GBM	6000	0
250	GBT	6000	0
250	RFB	6000	0
250	RFH	6000	0
500	MFP	6000	0
500	LMSS	6000	0
500	ENET	6000	0
500	GBM	6000	0
500	GBT	6000	0
500	RFB	6000	0
500	RFH	6000	0
1000	MFP	6000	0
1000	LMSS	6000	0
1000	ENET	6000	0
1000	GBM	6000	0
1000	GBT	6000	0
1000	RFB	6000	0
1000	RFH	6000	0

True inclusion frequency and true exclusion frequency

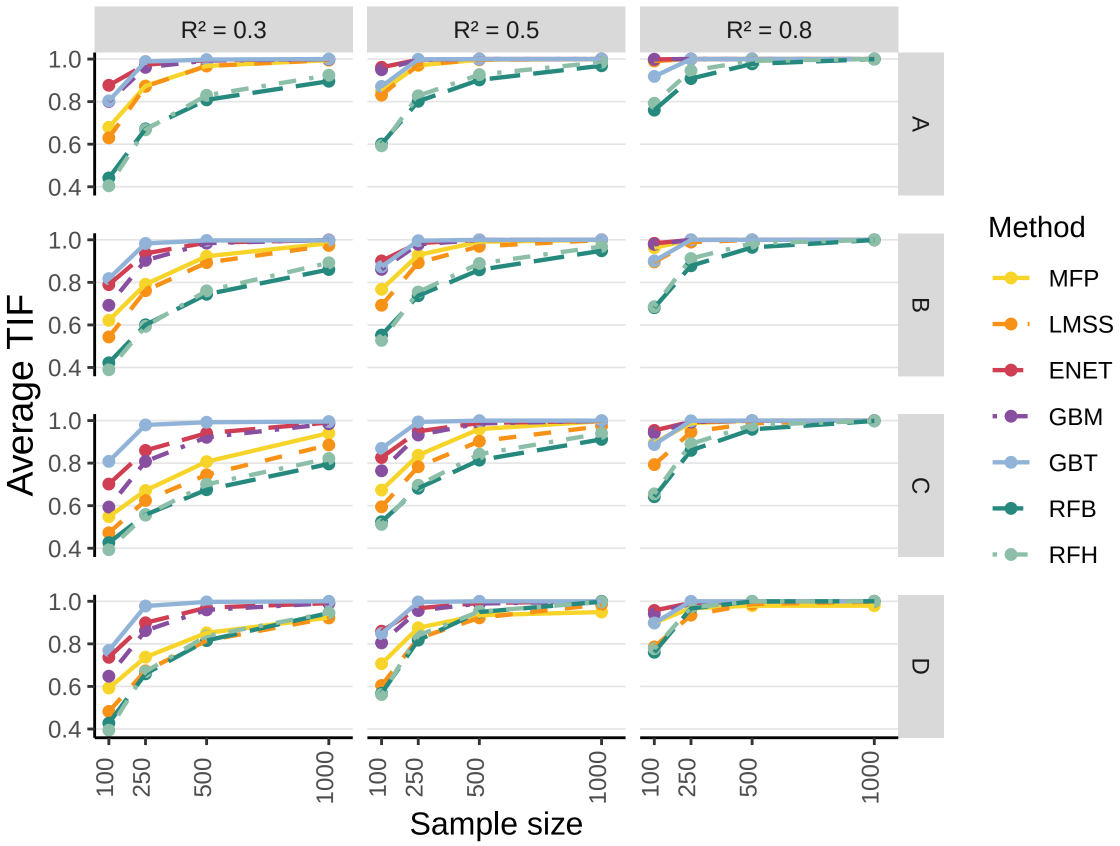

Figure S5: Average True Inclusion Frequency (TIF) of the 8 predictor variables across simulation repetitions.

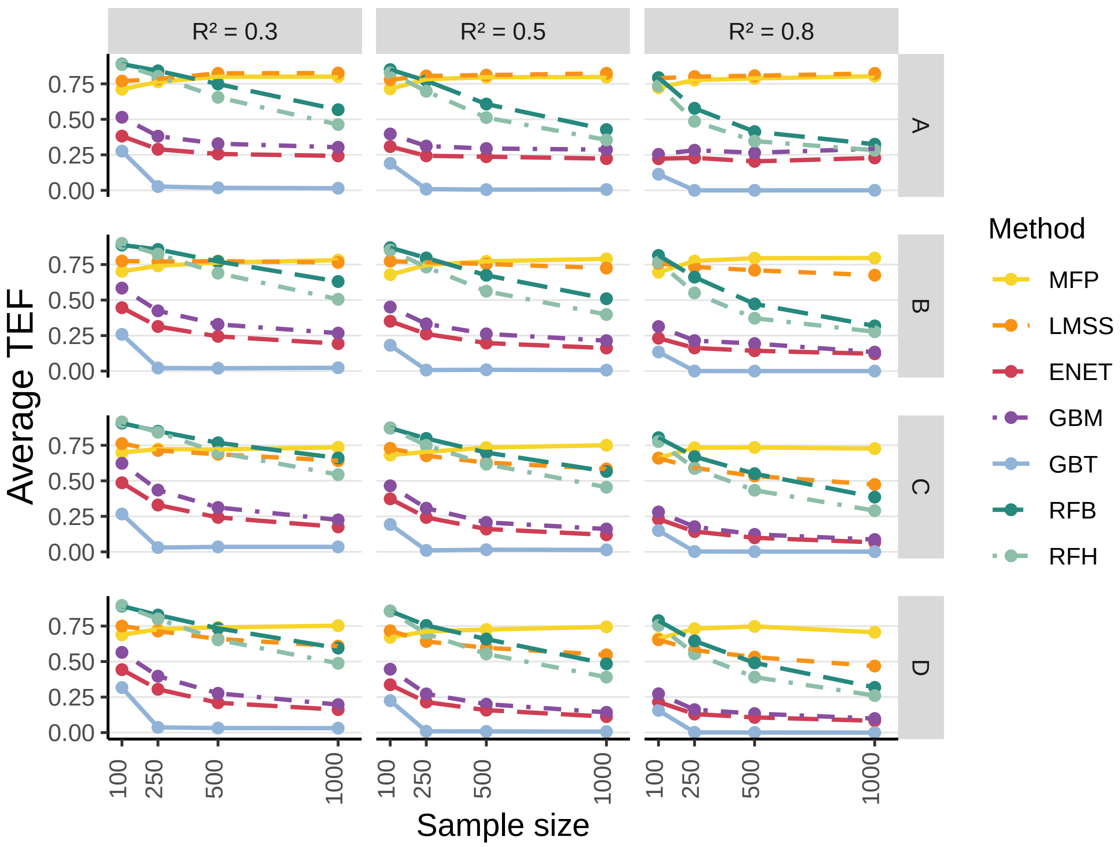

Figure S6: Average True Exclusion Frequency (TEF) of the 7 non-predictor variables across simulation repetitions.

Parsimony of the final models

Table S3: Model size by percentage of selected variables (SV) across all scenarios.

Method	SV [%]
MFP	57.5
LMSS	57.5
ENET	85.0
GBM	80.8
GBT	95.1
RFB	56.9
RFH	60.4

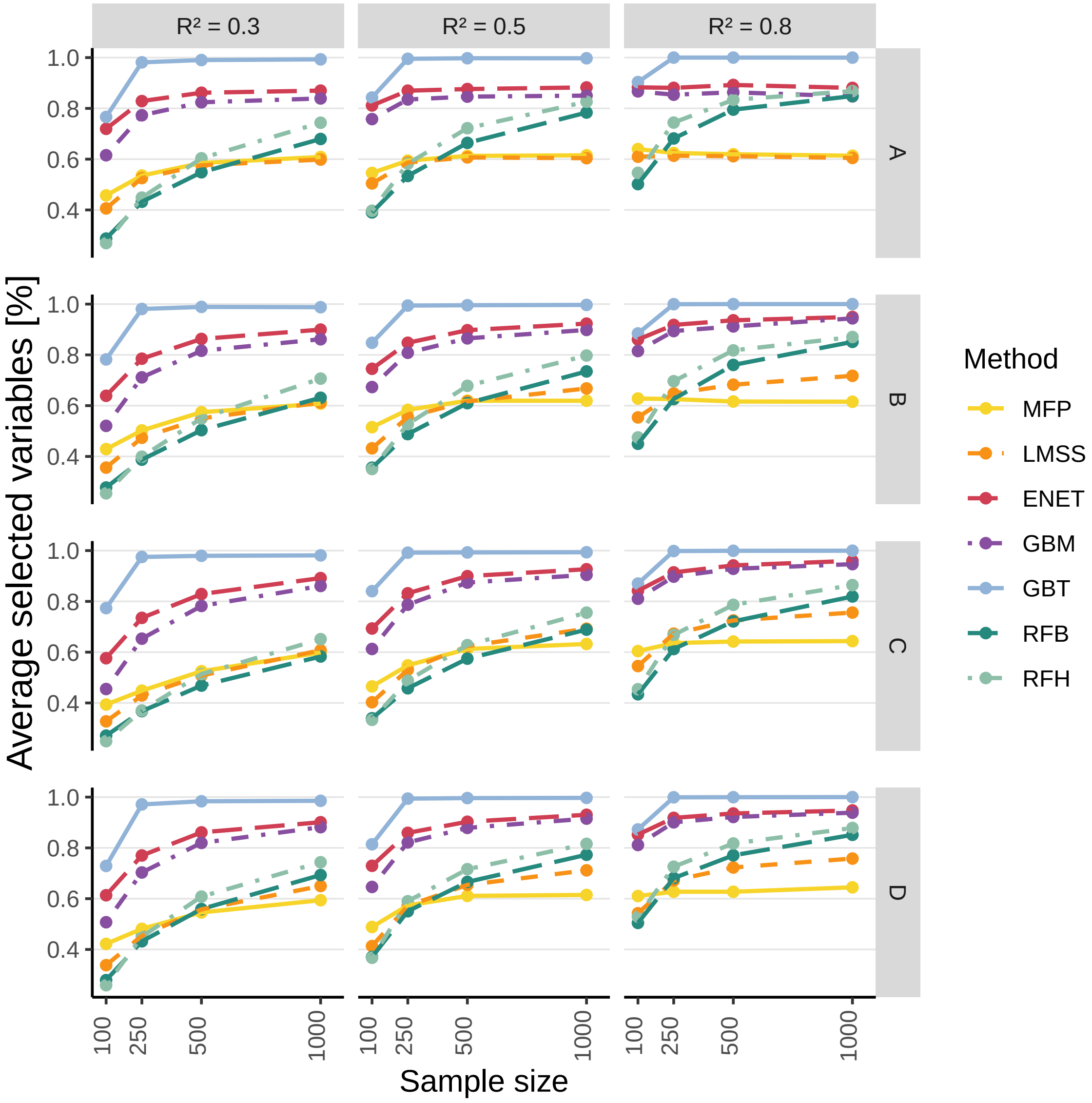

Figure S7: Average model size across simulation repetitions by percentage of selected variables from all available variables.

VIF of the most important predictors x5 and x3

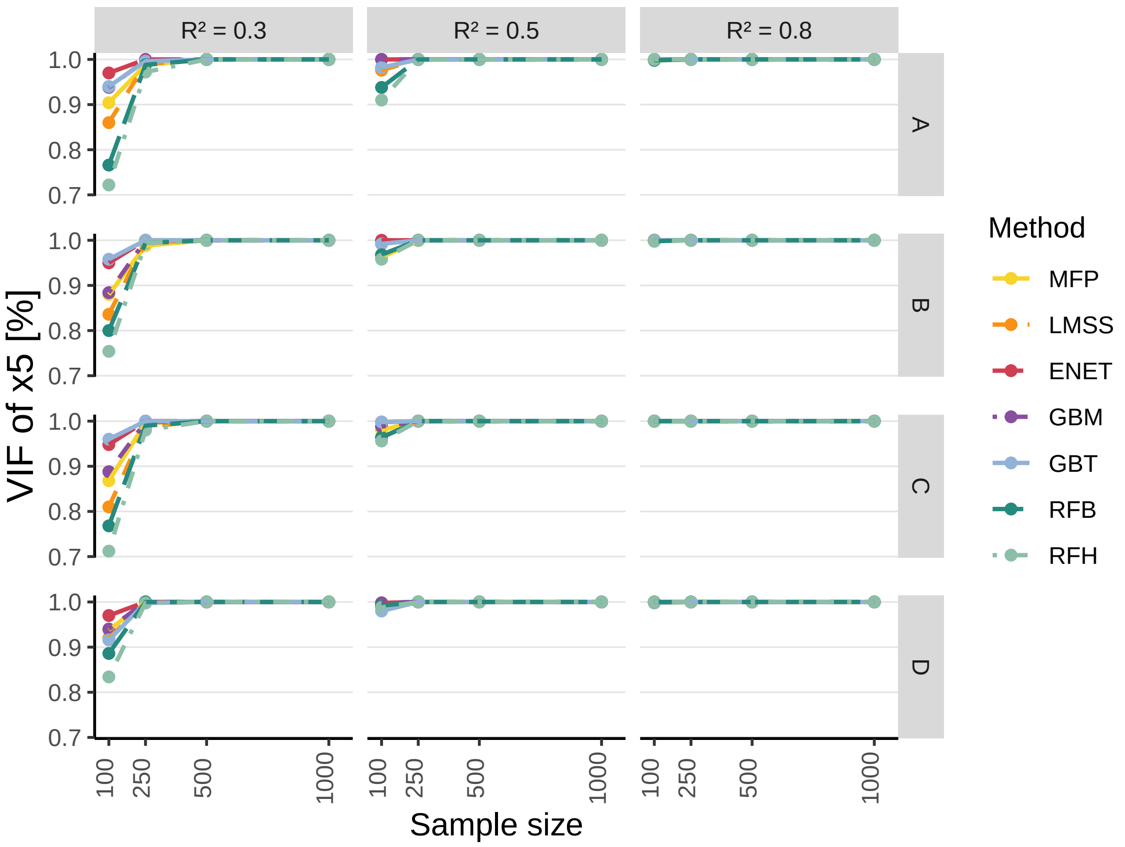

Figure S8: Variable Inclusion Frequency (VIF) across simulation repetitions of the most important predictor x5.

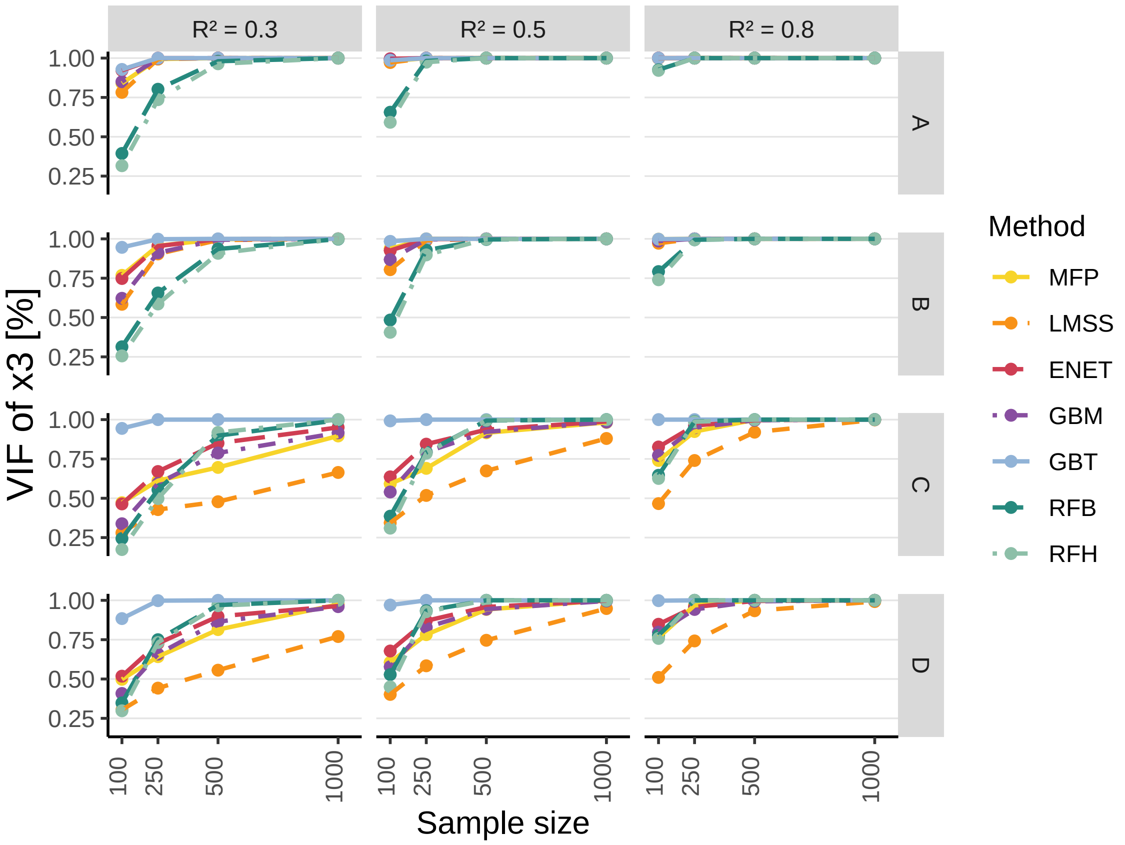

Figure S9: Variable Inclusion Frequency (VIF) across simulation repetitions of the second most important predictor x3.

VIF of predictors x11 and x8 involved in interaction effect

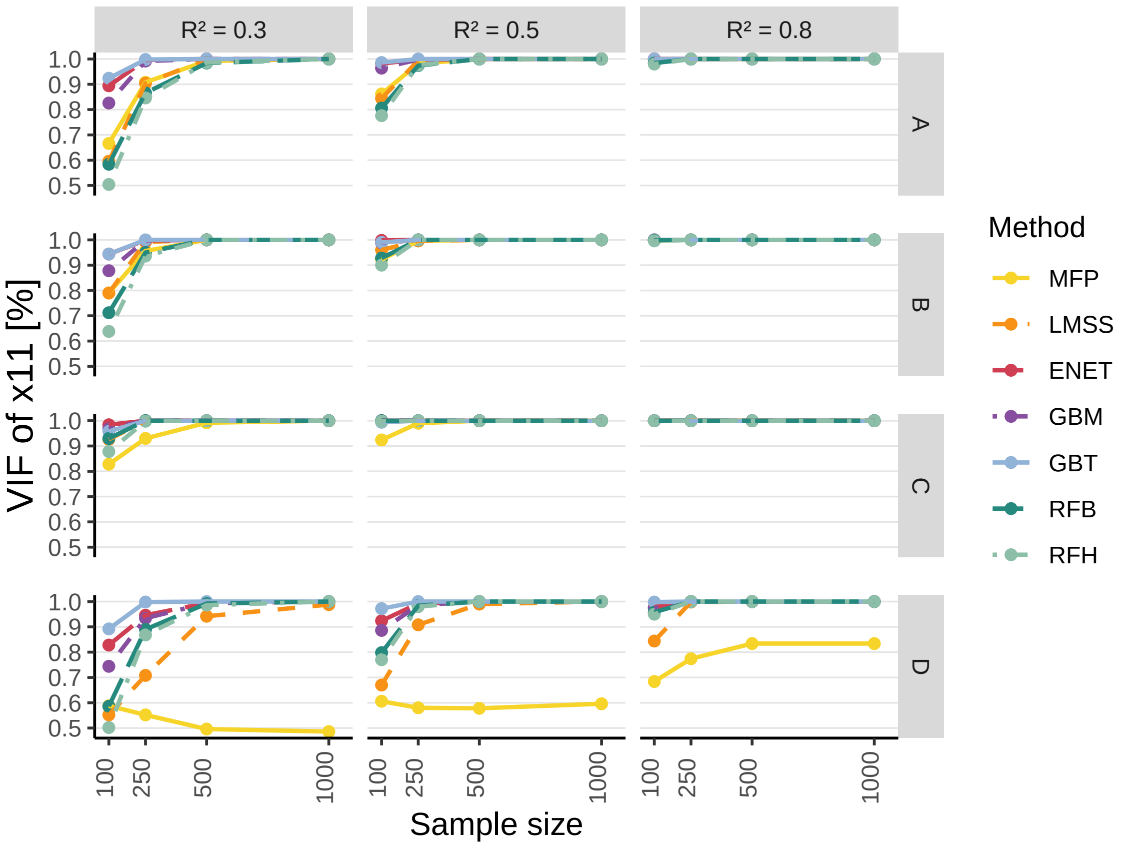

Figure S10: Variable Inclusion Frequency (VIF) across simulation repetitions of the predictor x11 which constitutes the interaction effect.

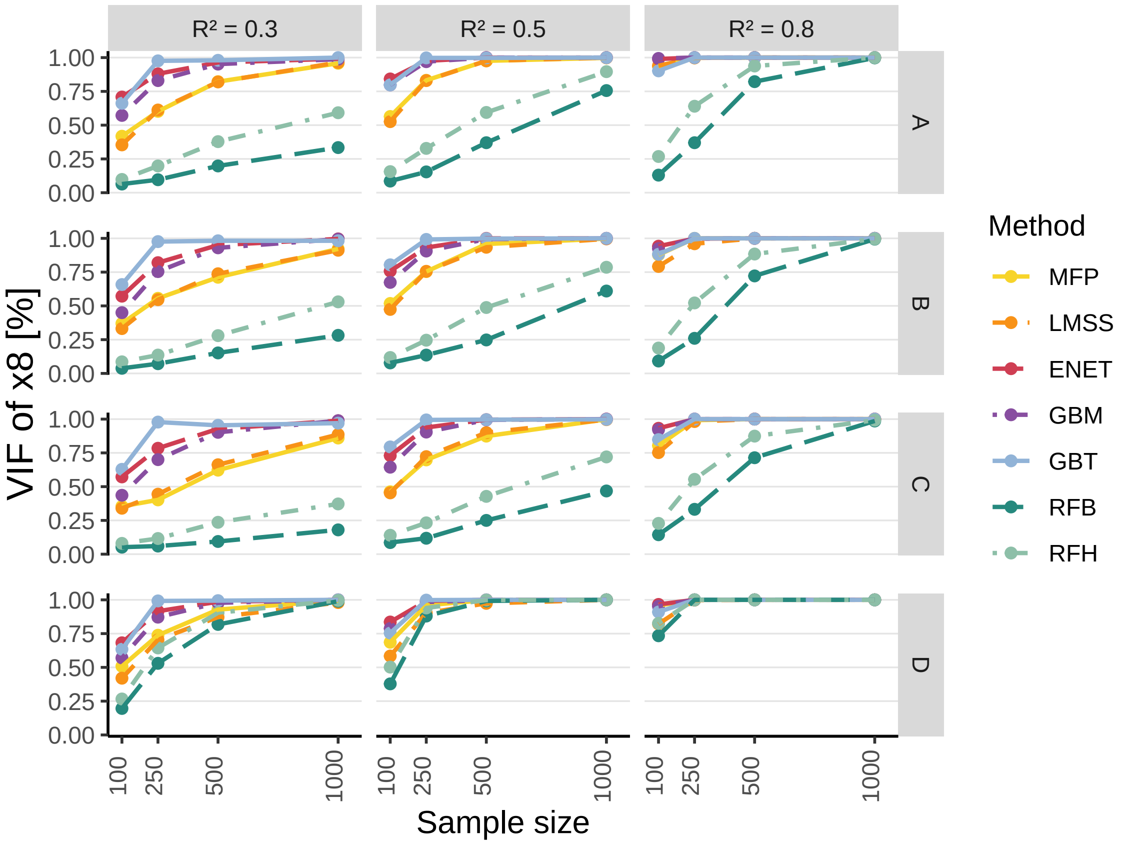

Figure S11: Variable Inclusion Frequency (VIF) across simulation repetitions of predictor x8 which contribute to the interaction effect.

Predictive performance

Table S4: Average RMSPE across simulation repetitions of the final models in the large test dataset for all scenarios.

Setting	R2	n	TOM	LMSS	ENET	GBM	GBT	RFB	RFH
A	0.3	100	0.945	0.999	0.970	0.975	1.055	1.035	1.042
A	0.3	250	0.917	0.936	0.930	0.931	0.995	0.981	0.983
A	0.3	500	0.908	0.915	0.915	0.915	0.963	0.959	0.959
A	0.3	1000	0.904	0.907	0.907	0.907	0.941	0.944	0.944
A	0.5	100	0.618	0.655	0.643	0.643	0.765	0.718	0.721
A	0.5	250	0.601	0.610	0.610	0.610	0.692	0.674	0.674
A	0.5	500	0.596	0.599	0.600	0.600	0.654	0.654	0.654
A	0.5	1000	0.593	0.594	0.595	0.595	0.630	0.640	0.640
A	0.8	100	0.307	0.319	0.320	0.320	0.509	0.455	0.454
A	0.8	250	0.298	0.302	0.303	0.303	0.413	0.411	0.411
A	0.8	500	0.295	0.297	0.297	0.297	0.360	0.387	0.388
A	0.8	1000	0.294	0.295	0.295	0.295	0.332	0.370	0.370
B	0.3	100	2.839	3.107	3.016	3.029	3.153	3.081	3.106
B	0.3	250	2.756	2.928	2.907	2.911	2.982	2.944	2.949
B	0.3	500	2.729	2.869	2.861	2.862	2.888	2.881	2.880
B	0.3	1000	2.717	2.841	2.839	2.839	2.827	2.836	2.834
B	0.5	100	1.869	2.160	2.114	2.120	2.291	2.144	2.153
B	0.5	250	1.816	2.025	2.016	2.016	2.082	2.027	2.027
B	0.5	500	1.798	1.986	1.983	1.983	1.973	1.969	1.967
B	0.5	1000	1.790	1.965	1.965	1.966	1.903	1.928	1.928
B	0.8	100	0.941	1.327	1.310	1.312	1.555	1.361	1.365
B	0.8	250	0.912	1.237	1.236	1.237	1.257	1.230	1.231
B	0.8	500	0.903	1.212	1.213	1.213	1.104	1.167	1.167
B	0.8	1000	0.899	1.201	1.201	1.202	1.017	1.114	1.114
C	0.3	100	3.382	3.602	3.513	3.520	3.721	3.610	3.632
C	0.3	250	3.285	3.428	3.397	3.400	3.510	3.473	3.476
C	0.3	500	3.254	3.367	3.352	3.353	3.414	3.408	3.406
C	0.3	1000	3.238	3.330	3.325	3.325	3.353	3.361	3.360
C	0.5	100	2.211	2.439	2.380	2.386	2.634	2.449	2.456
C	0.5	250	2.140	2.305	2.284	2.285	2.407	2.342	2.340
C	0.5	500	2.120	2.257	2.249	2.249	2.291	2.278	2.276
C	0.5	1000	2.110	2.230	2.229	2.229	2.224	2.236	2.235
C	0.8	100	1.107	1.413	1.381	1.384	1.739	1.488	1.490
C	0.8	250	1.073	1.310	1.305	1.305	1.422	1.361	1.361
C	0.8	500	1.064	1.282	1.281	1.281	1.264	1.301	1.301
C	0.8	1000	1.059	1.269	1.269	1.269	1.179	1.255	1.256
D	0.3	100	2.724	3.005	2.917	2.930	3.072	3.001	3.018
D	0.3	250	2.646	2.855	2.824	2.828	2.904	2.850	2.848
D	0.3	500	2.621	2.799	2.786	2.787	2.818	2.788	2.784
D	0.3	1000	2.609	2.768	2.764	2.765	2.753	2.737	2.737
D	0.5	100	1.770	2.110	2.055	2.063	2.240	2.086	2.089
D	0.5	250	1.719	1.988	1.969	1.970	2.039	1.956	1.953
D	0.5	500	1.703	1.942	1.937	1.938	1.930	1.888	1.889
D	0.5	1000	1.696	1.920	1.920	1.920	1.848	1.844	1.845
D	0.8	100	0.890	1.363	1.329	1.334	1.560	1.346	1.345
D	0.8	250	0.863	1.264	1.258	1.258	1.281	1.202	1.203
D	0.8	500	0.855	1.237	1.236	1.236	1.120	1.128	1.130
D	0.8	1000	0.851	1.223	1.223	1.223	1.021	1.069	1.070

Table S5: Monte Carlo standard error of RMSPE for all scenarios.

Setting	R2	n	TOM	LMSS	ENET	GBM	GBT	RFB	RFH
A	0.3	100	0.0010	0.0017	0.0012	0.0013	0.0020	0.0017	0.0019
A	0.3	250	0.0004	0.0006	0.0005	0.0005	0.0010	0.0007	0.0007
A	0.3	500	0.0002	0.0003	0.0002	0.0002	0.0005	0.0004	0.0004
A	0.3	1000	0.0001	0.0001	0.0001	0.0001	0.0003	0.0002	0.0002
A	0.5	100	0.0006	0.0013	0.0009	0.0009	0.0018	0.0010	0.0010
A	0.5	250	0.0002	0.0004	0.0003	0.0003	0.0007	0.0005	0.0006
A	0.5	500	0.0001	0.0002	0.0002	0.0002	0.0004	0.0003	0.0003
A	0.5	1000	0.0001	0.0001	0.0001	0.0001	0.0003	0.0002	0.0002
A	0.8	100	0.0003	0.0006	0.0005	0.0005	0.0012	0.0009	0.0009
A	0.8	250	0.0001	0.0002	0.0002	0.0002	0.0006	0.0005	0.0005
A	0.8	500	0.0001	0.0001	0.0001	0.0001	0.0003	0.0003	0.0003
A	0.8	1000	0.0000	0.0000	0.0000	0.0000	0.0002	0.0002	0.0002
B	0.3	100	0.0030	0.0048	0.0033	0.0035	0.0069	0.0051	0.0063
B	0.3	250	0.0011	0.0018	0.0014	0.0015	0.0030	0.0020	0.0021
B	0.3	500	0.0005	0.0009	0.0008	0.0008	0.0016	0.0012	0.0013
B	0.3	1000	0.0003	0.0005	0.0004	0.0004	0.0009	0.0007	0.0007
B	0.5	100	0.0018	0.0036	0.0026	0.0029	0.0057	0.0030	0.0029
B	0.5	250	0.0007	0.0014	0.0011	0.0011	0.0021	0.0016	0.0017
B	0.5	500	0.0004	0.0007	0.0006	0.0006	0.0013	0.0010	0.0009
B	0.5	1000	0.0002	0.0003	0.0003	0.0003	0.0008	0.0006	0.0006
B	0.8	100	0.0010	0.0024	0.0020	0.0021	0.0040	0.0023	0.0025
B	0.8	250	0.0004	0.0009	0.0007	0.0008	0.0019	0.0014	0.0014
B	0.8	500	0.0002	0.0004	0.0004	0.0004	0.0011	0.0009	0.0009
B	0.8	1000	0.0001	0.0002	0.0002	0.0002	0.0006	0.0006	0.0006
C	0.3	100	0.0035	0.0052	0.0039	0.0041	0.0086	0.0053	0.0062
C	0.3	250	0.0013	0.0019	0.0016	0.0016	0.0028	0.0023	0.0025
C	0.3	500	0.0007	0.0010	0.0009	0.0009	0.0017	0.0013	0.0014
C	0.3	1000	0.0003	0.0006	0.0004	0.0005	0.0010	0.0008	0.0008
C	0.5	100	0.0025	0.0040	0.0030	0.0031	0.0059	0.0029	0.0030
C	0.5	250	0.0008	0.0014	0.0012	0.0012	0.0024	0.0017	0.0017
C	0.5	500	0.0004	0.0008	0.0006	0.0006	0.0013	0.0010	0.0010
C	0.5	1000	0.0002	0.0004	0.0003	0.0003	0.0008	0.0006	0.0006
C	0.8	100	0.0011	0.0025	0.0020	0.0021	0.0043	0.0025	0.0025
C	0.8	250	0.0004	0.0009	0.0008	0.0008	0.0020	0.0014	0.0013
C	0.8	500	0.0002	0.0004	0.0004	0.0004	0.0011	0.0008	0.0008
C	0.8	1000	0.0001	0.0002	0.0002	0.0002	0.0007	0.0006	0.0006
D	0.3	100	0.0028	0.0044	0.0032	0.0034	0.0071	0.0056	0.0064
D	0.3	250	0.0011	0.0016	0.0013	0.0014	0.0030	0.0021	0.0022
D	0.3	500	0.0005	0.0009	0.0007	0.0007	0.0015	0.0013	0.0012
D	0.3	1000	0.0003	0.0005	0.0004	0.0004	0.0009	0.0007	0.0007
D	0.5	100	0.0018	0.0031	0.0025	0.0027	0.0052	0.0030	0.0031
D	0.5	250	0.0007	0.0013	0.0010	0.0010	0.0021	0.0016	0.0015
D	0.5	500	0.0003	0.0007	0.0006	0.0006	0.0013	0.0010	0.0009
D	0.5	1000	0.0002	0.0003	0.0003	0.0003	0.0008	0.0006	0.0006
D	0.8	100	0.0010	0.0023	0.0018	0.0020	0.0035	0.0026	0.0026
D	0.8	250	0.0004	0.0009	0.0007	0.0007	0.0018	0.0013	0.0013
D	0.8	500	0.0002	0.0004	0.0004	0.0004	0.0012	0.0009	0.0009
D	0.8	1000	0.0001	0.0002	0.0002	0.0002	0.0007	0.0006	0.0006

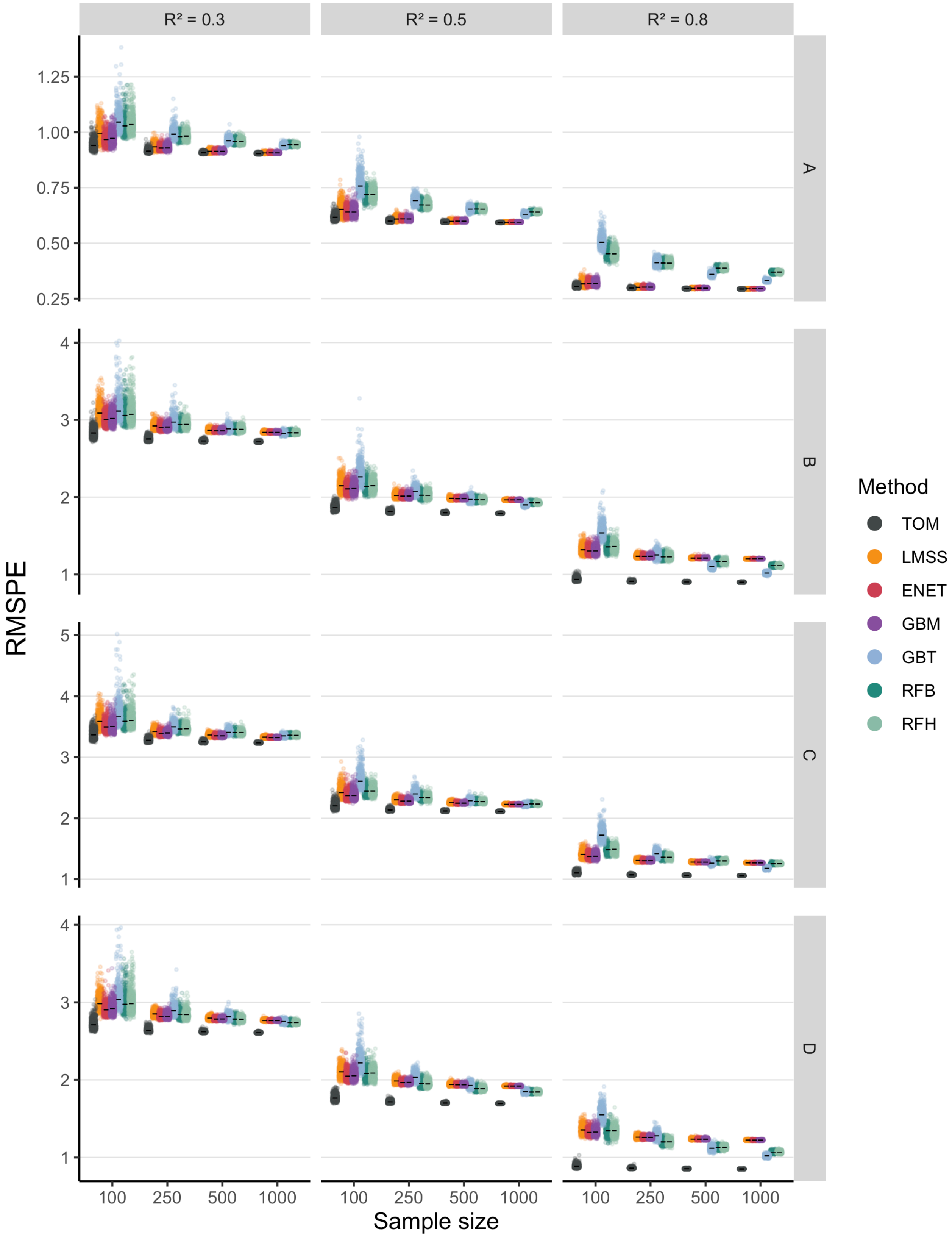

Figure S12: Root mean squared prediction error (RMSPE) of the final models in the large test dataset, with the average across simulation repetitions indicated by the short horizontal lines.

RMSPE and model size

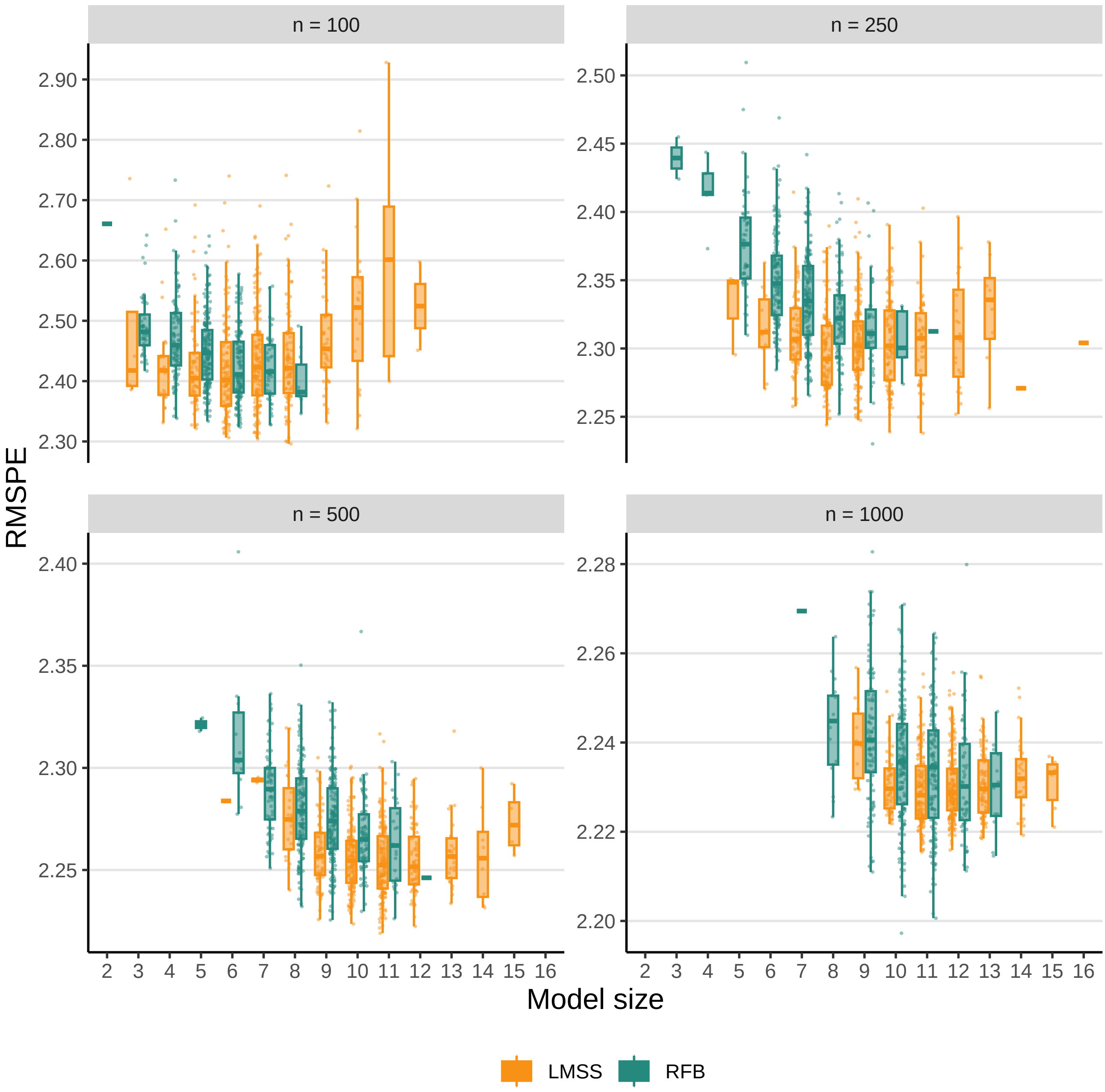

Figure S13: Root mean squared prediction error (RMSPE) in dependence of the model size by the number of selected variables for the scenarios of setting C and $R^2 = 0.5$ comparing LMSS and RFB.

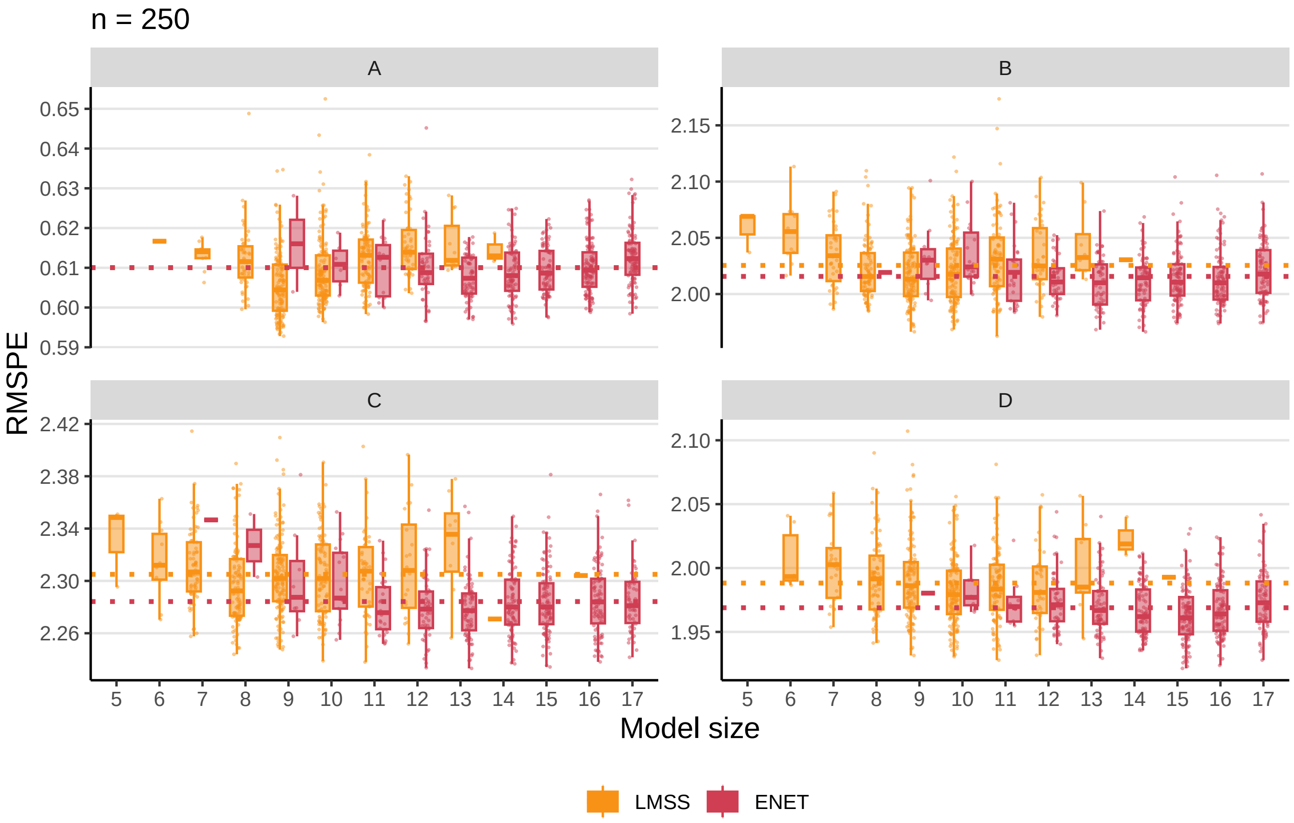

Figure S14: RMSPE in dependence of the model size by the number of selected variables for the scenarios with 250 observations and $R^2 = 0.5$ comparing LMSS and ENET. The dotted lines represent the average RMSPE of the method in the scenario, respectively.

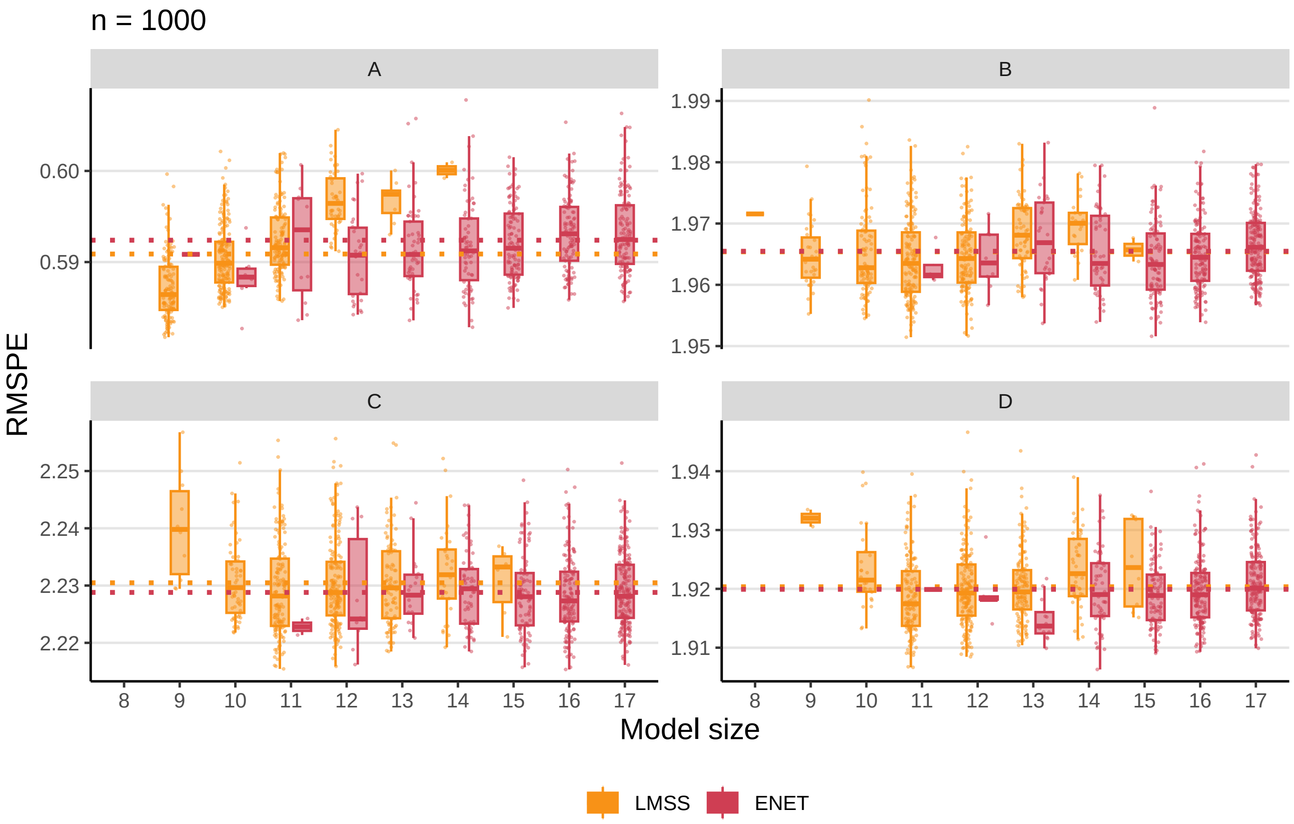

Figure S15: RMSPE in dependence of the model size by the number of selected variables for the scenarios with 1000 observations and $R^2 = 0.5$ comparing LMSS and ENET. The dotted lines represent the average RMSPE of the method in the scenario, respectively.

Calibration slope

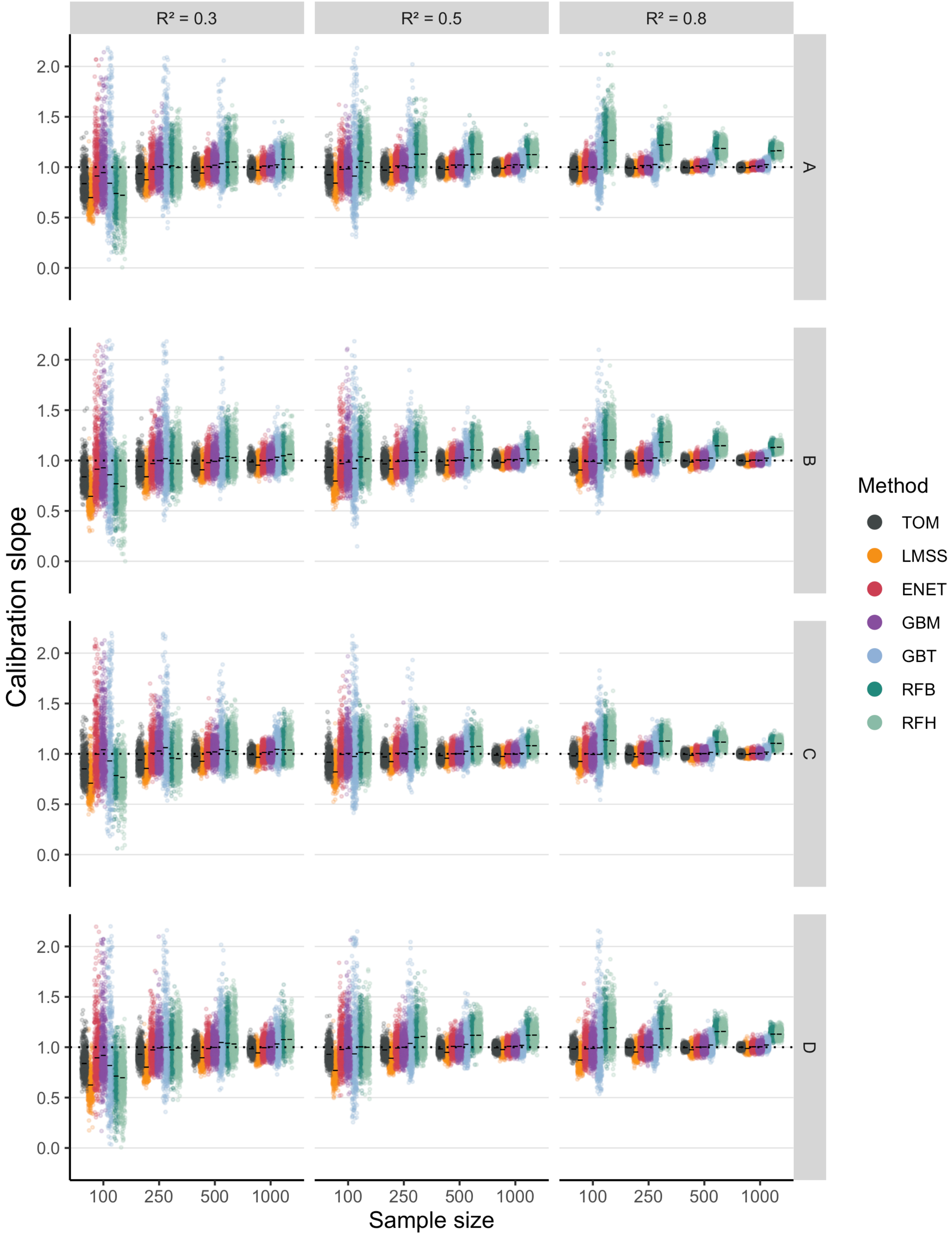

Figure S16: Calibration slope of the final models in the large test dataset, with the median across simulation repetitions indicated by the short horizontal lines.

MAD of calibration slope

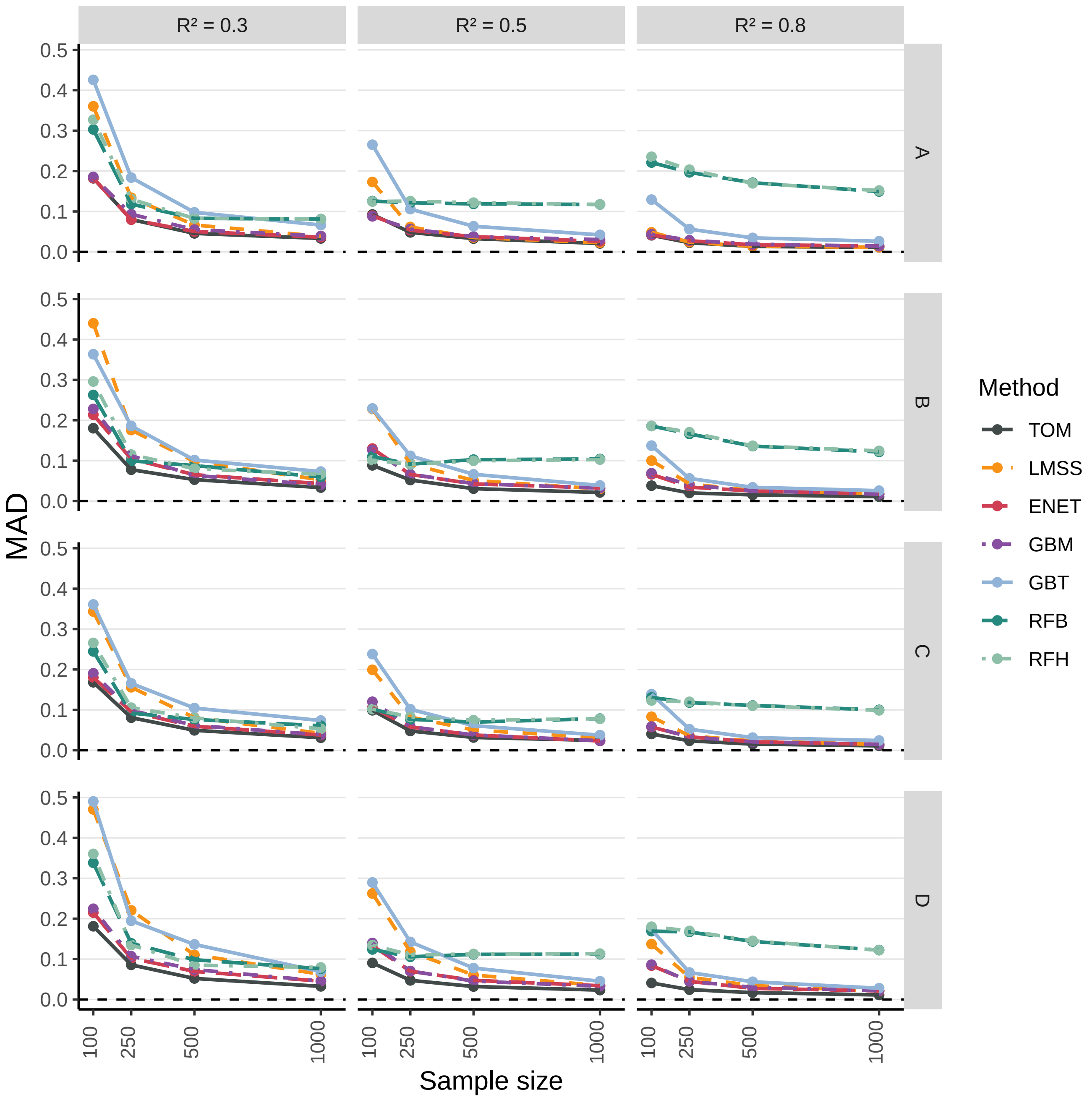

Figure S17: Median absolute deviation (MAD) of the log(slope) of the final models in the test dataset, showing the variability in the calibration slope.

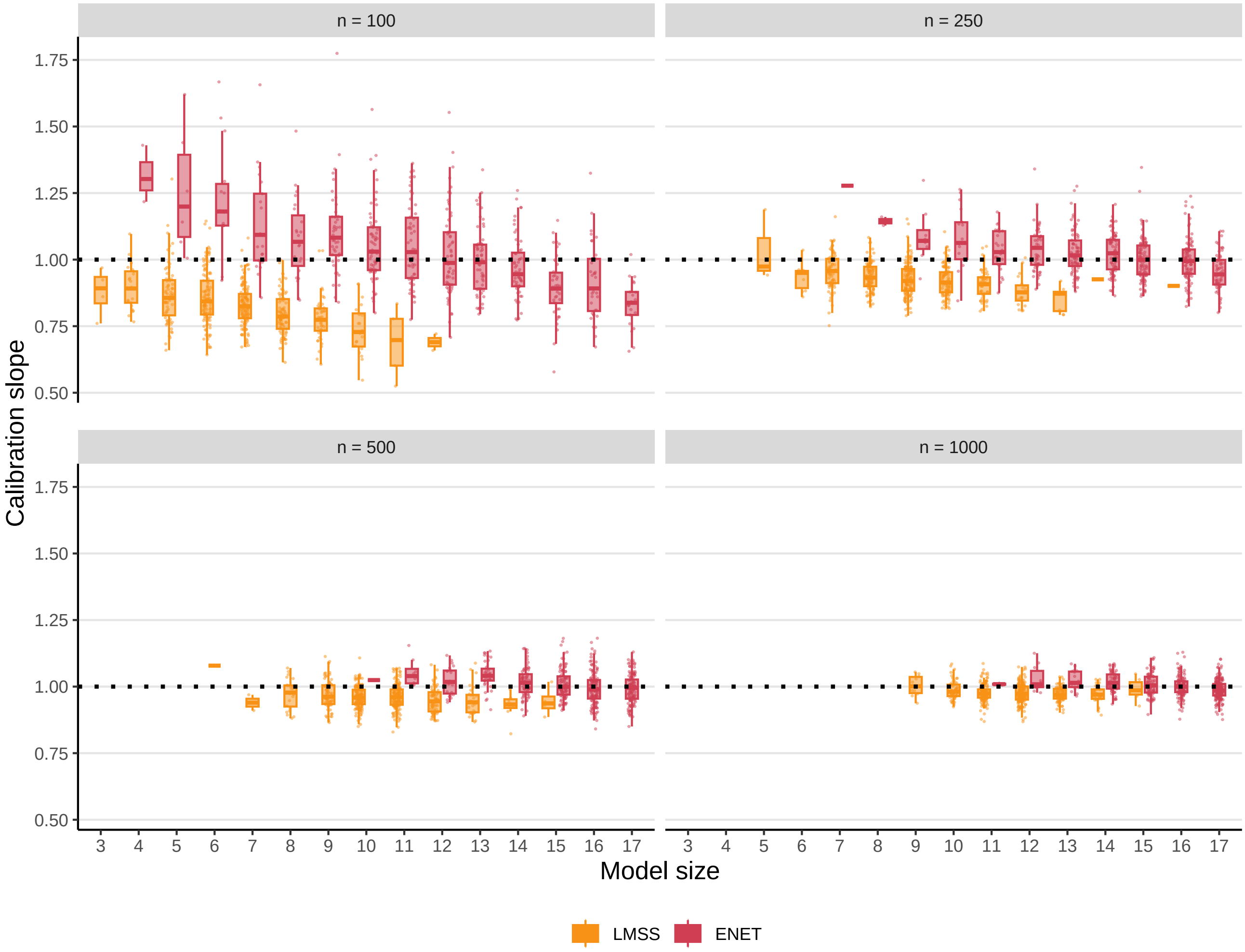

Figure S18: Calibration slope in dependence of the model size by the number of selected variables for the scenarios of complexity level C and $R^2 = 0.5$ comparing LMSS and ENET.

Oracle models

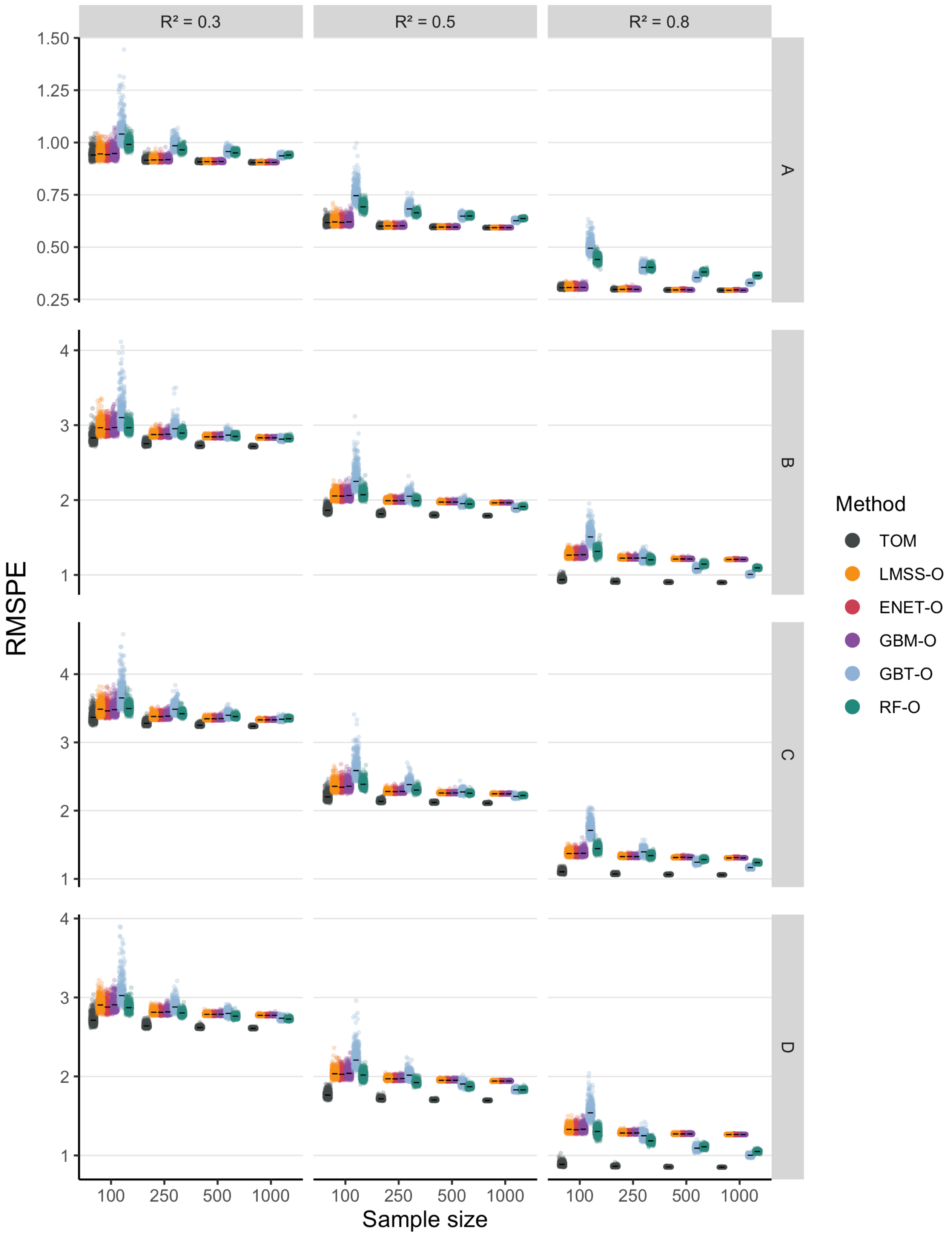

Figure S19: Root mean squared prediction error (RMSPE) of the oracle models in the large test dataset, with the average across simulation repetitions indicated by the short horizontal lines.

Comparison to oracle models

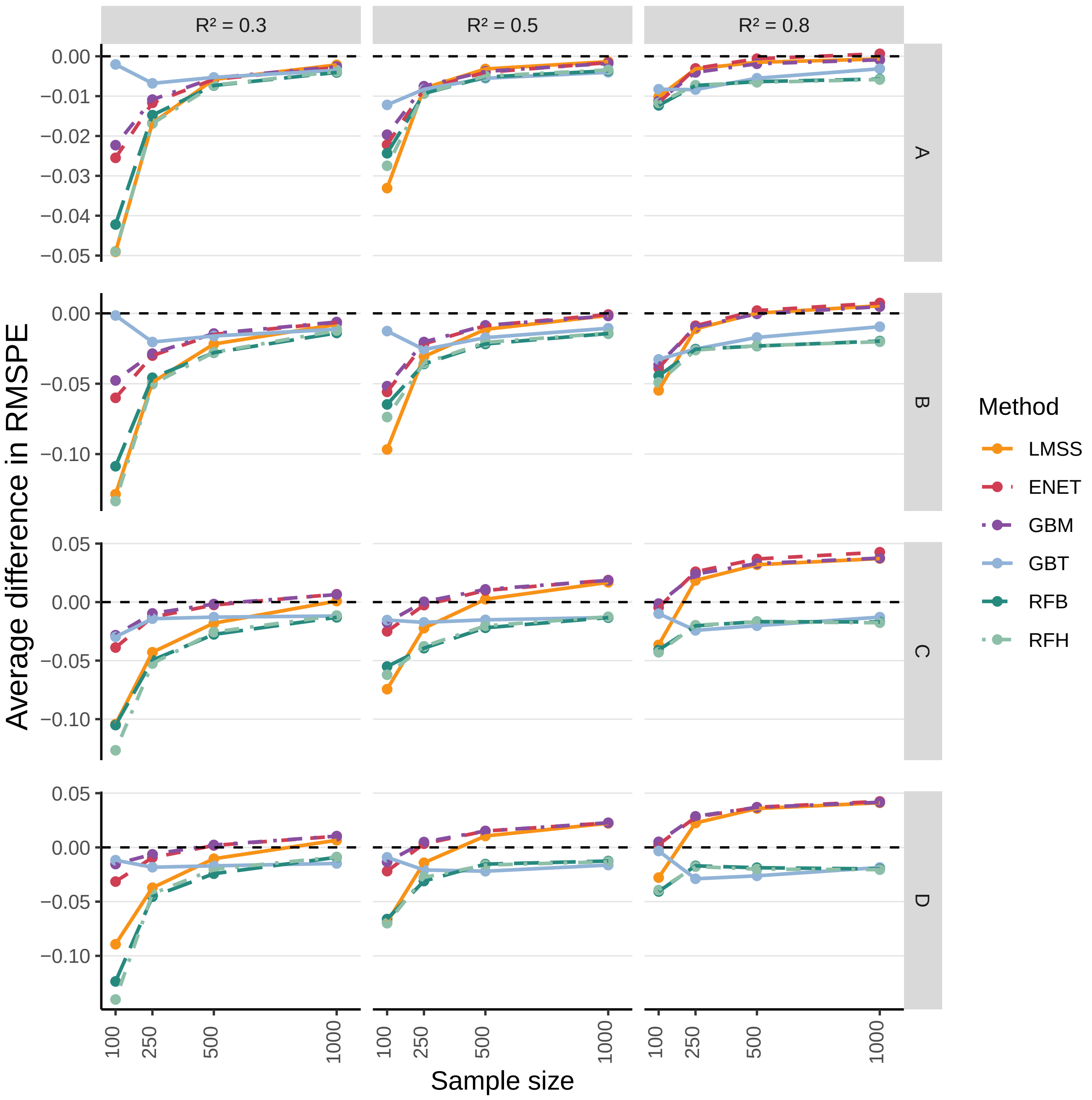

Figure S20: Deviation of RMSPE between the finally developed models and their corresponding oracle models. A negative value indicates that the final model revealed a higher (worse) RMSPE than its oracle model. A value above 0 indicates that the final model obtained a lower (better) RMSPE than its oracle model.

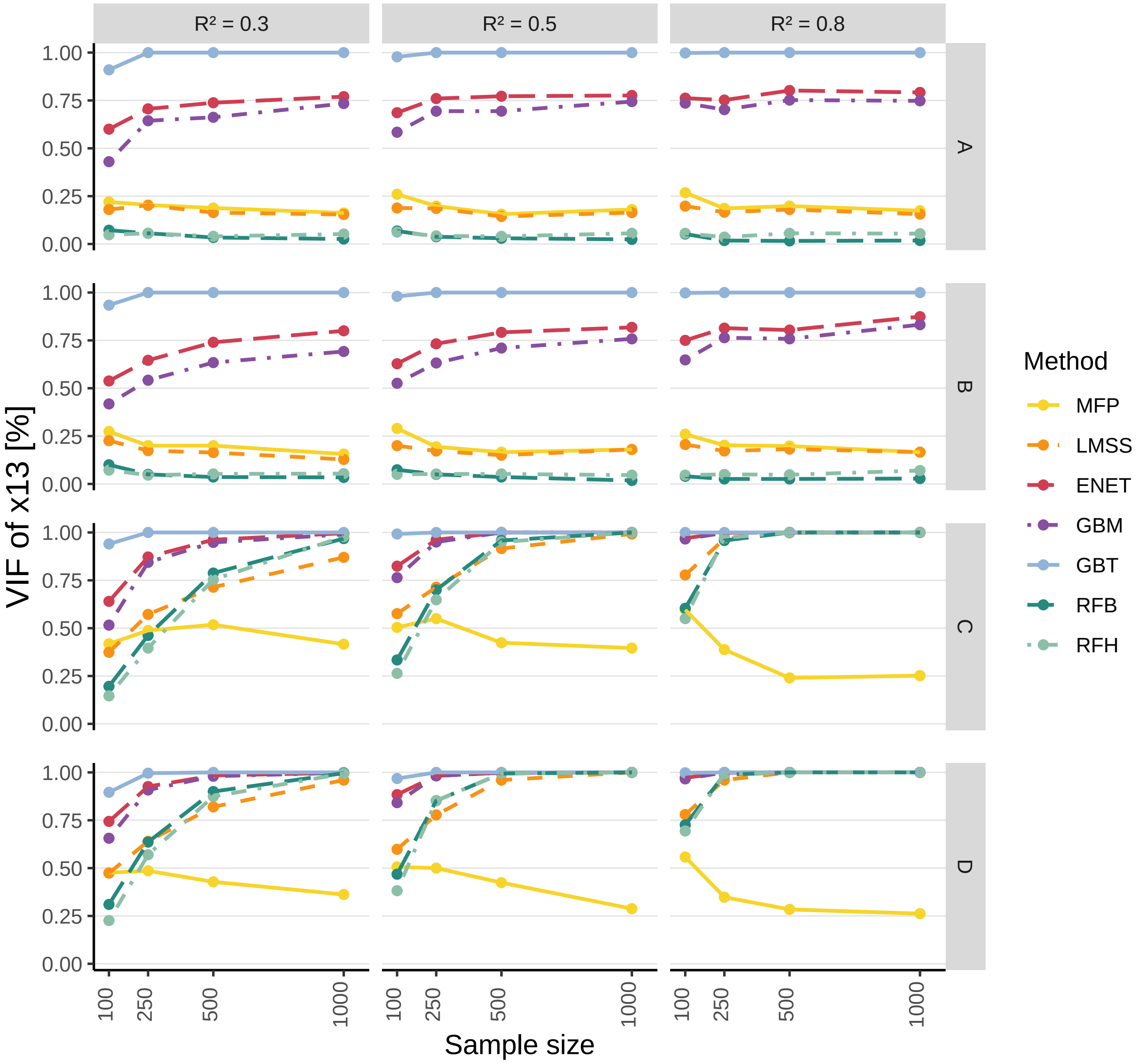

Figure S21: Variable Inclusion Frequency (VIF) across simulation repetitions of non-predictor variable x13.

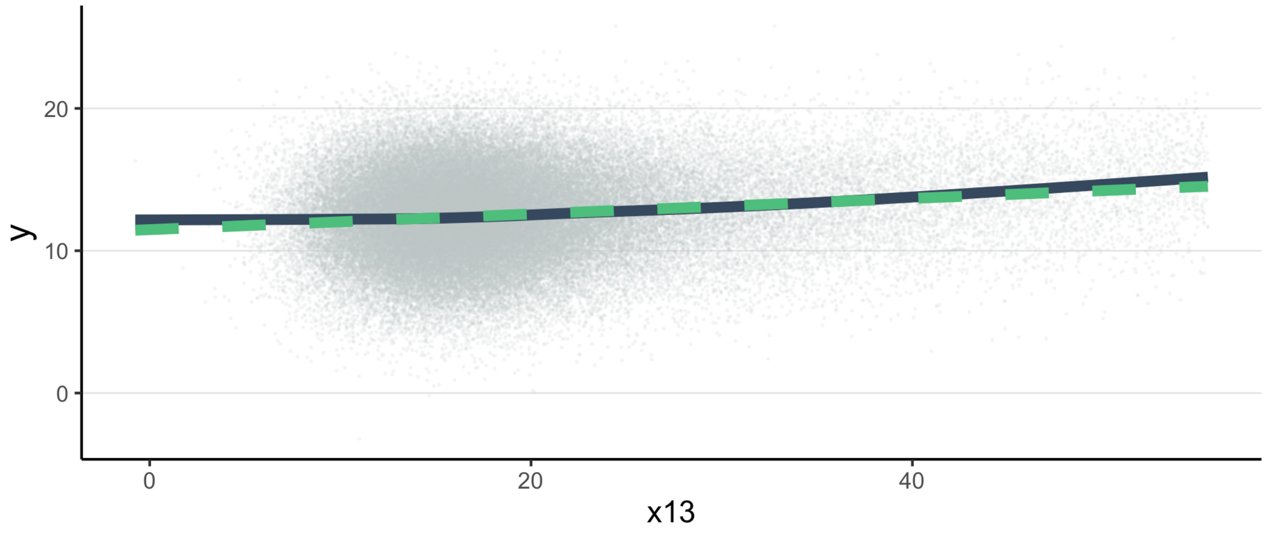

Figure S22: Scatter plot of non-predictor x13 and the outcome variable (y) in complexity level C and R^2 of 0.5.

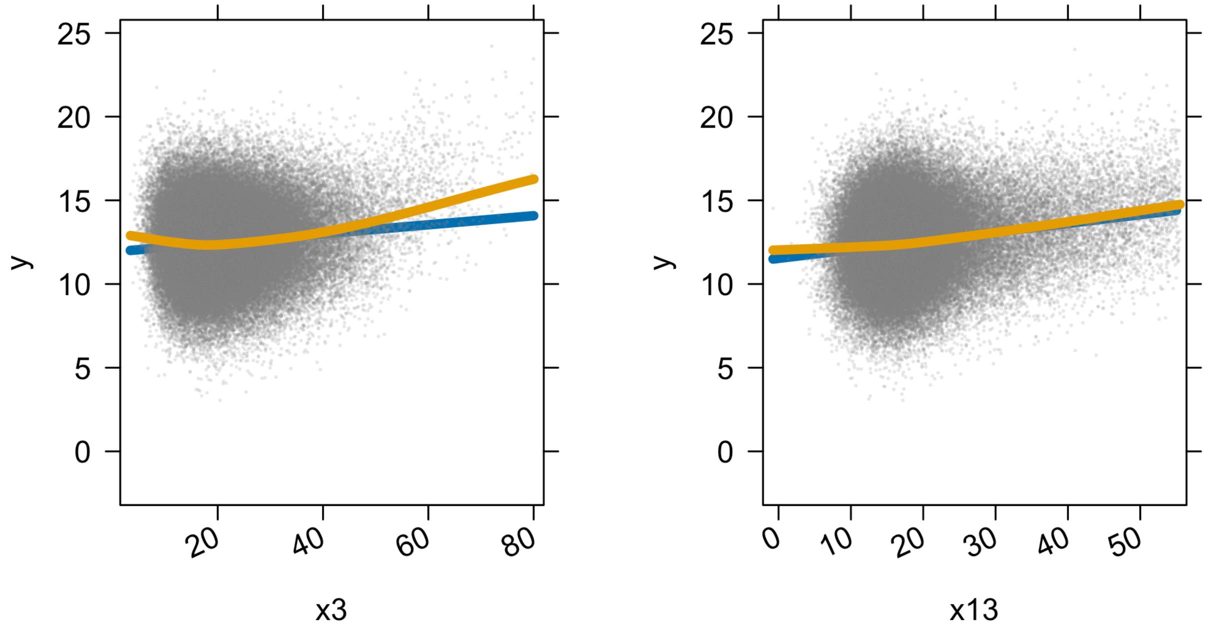

Figure S23: Partial residual plots of predictor x3 and non-predictor x13 based on a linear regression model containing the predictor variables and x13 with linear functional forms. The model was fitted on a dataset with 100000 observations in complexity level C and R^2 of 0.5.

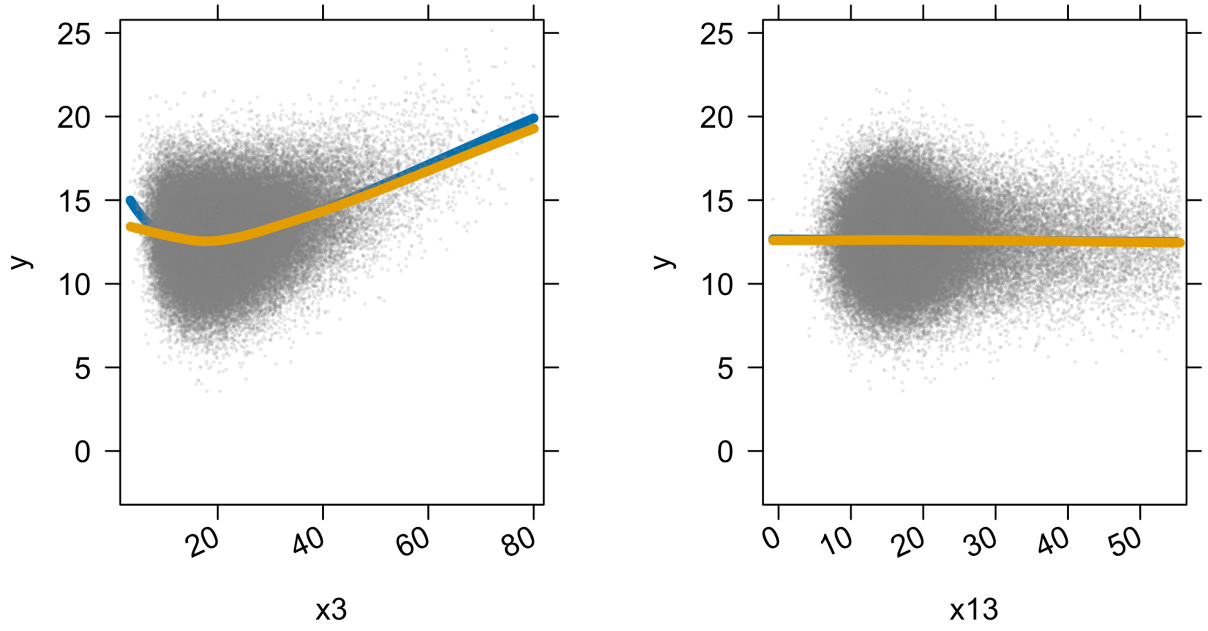

Figure S24: Partial residual plots of predictor x3 and non-predictor x13 based on a linear regression model containing the predictor variables with their true functional forms and x13. The model was fitted on a dataset with 100000 observations in complexity level C and R^2 of 0.5.

Size-restricted models

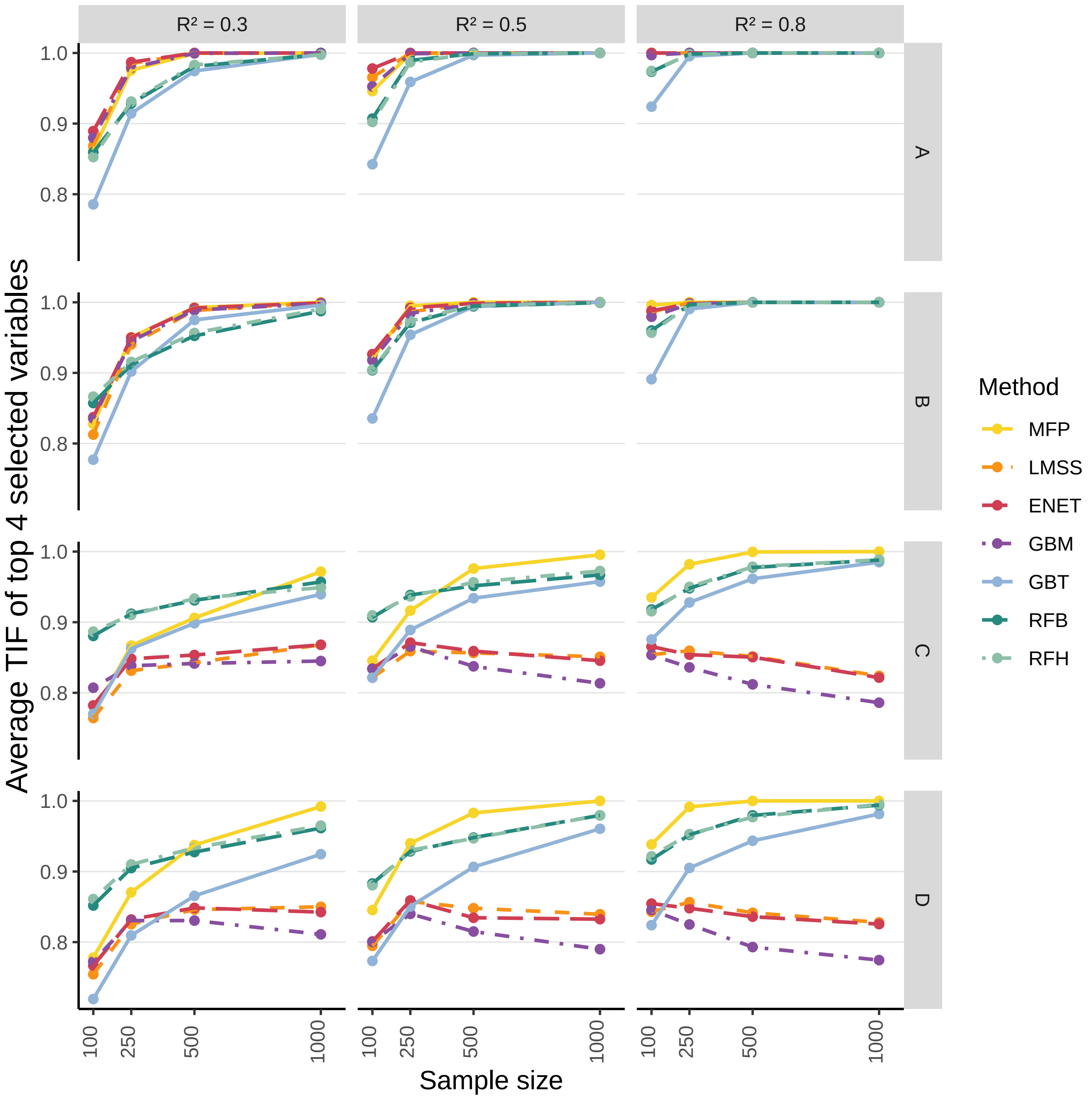

Figure S25: Average true inclusion frequency (TIF) of the size-restricted models across simulation repetitions.

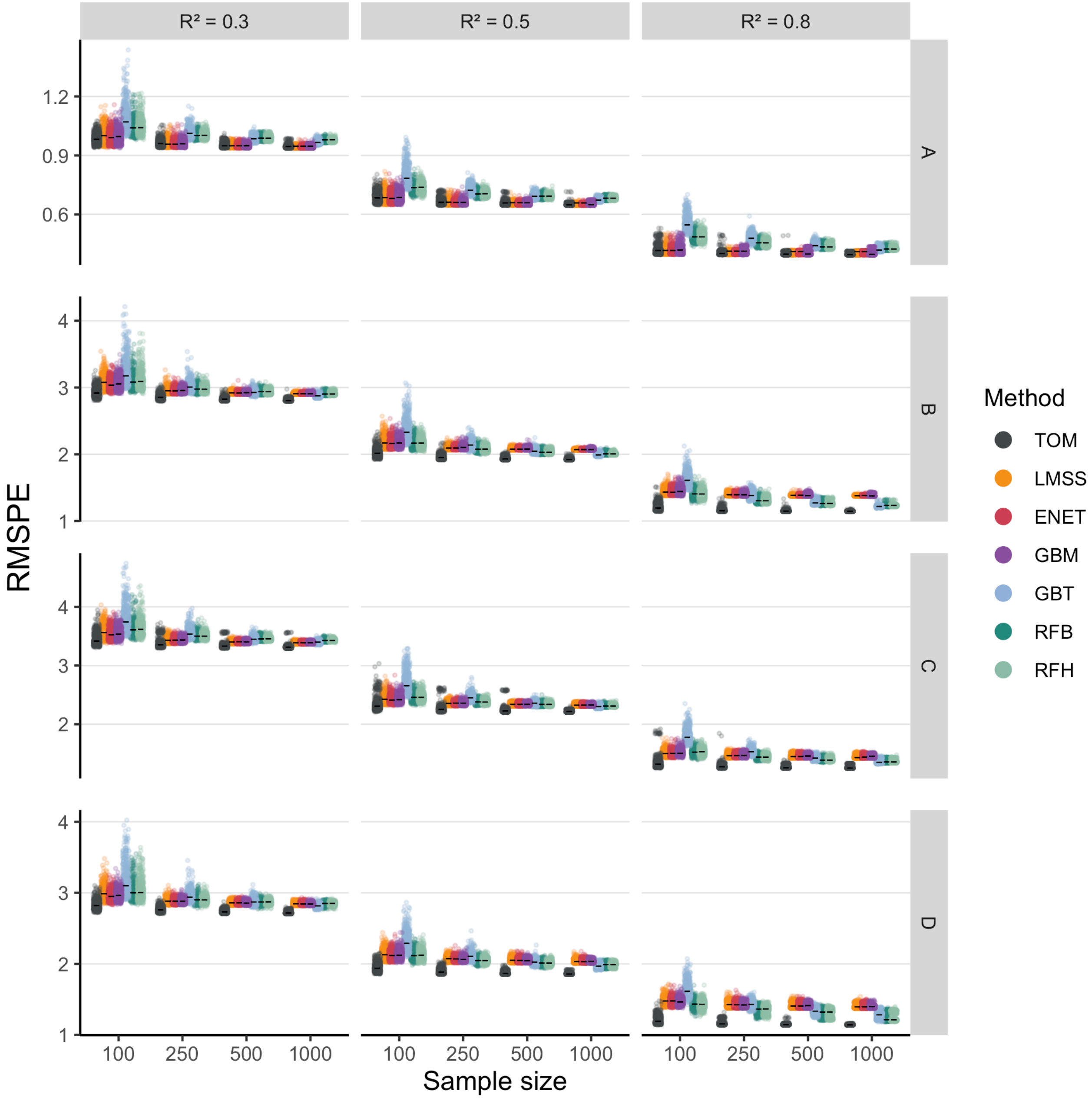

Figure S26: Root mean squared prediction error (RMSPE) of the size-restricted models in the large test dataset, with the average across simulation repetitions indicated by the short horizontal lines.

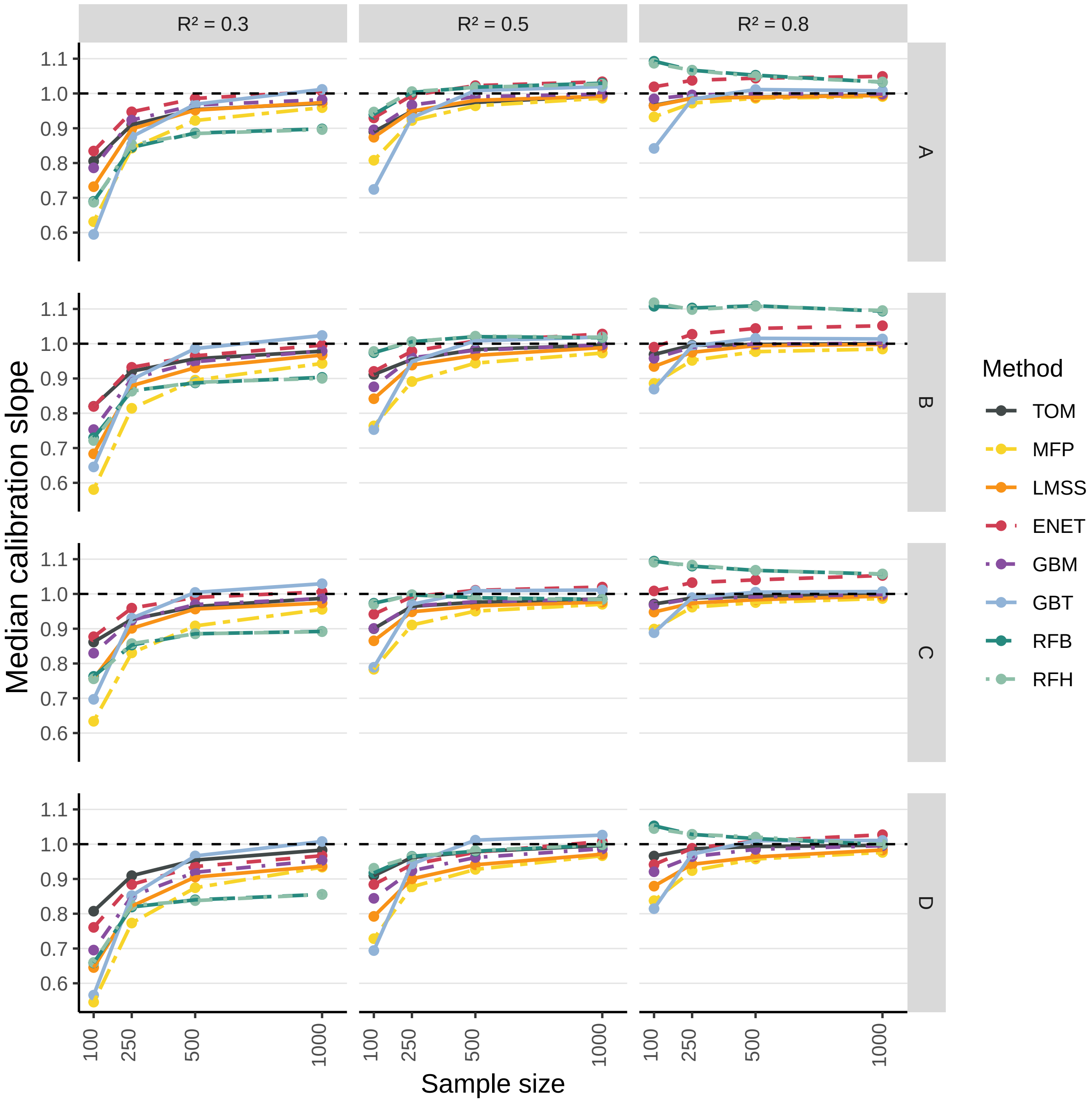

Figure S27: Median calibration slope of the size-restricted models in the large test dataset across simulation repetitions.

Running time

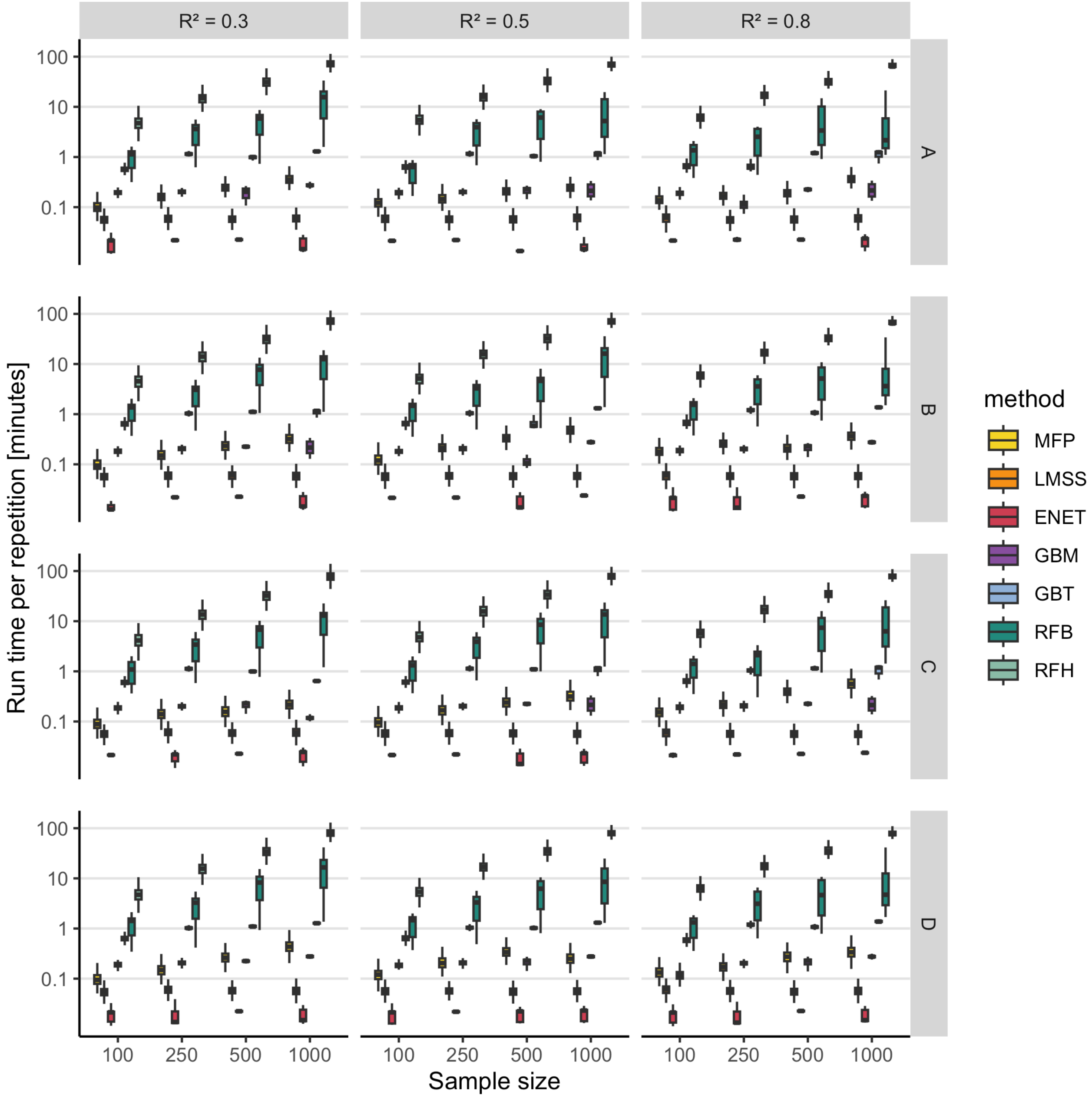

Figure S28: Computational time in minutes on a logarithmic scale.

Real data example

Table S6: Description of the outcome and candidate variables of the real data example.

Characteristic	N = 779
Blood loss (log-transformed) [ml]	
Mean (SD)	8.00 (0.89)
Median (Q1, Q3)	8.01 (7.38, 8.61)
Min, Max	5.01, 10.90
Age (recipient) [y]	
Mean (SD)	52 (10)
Median (Q1, Q3)	54 (46, 60)
Min, Max	19, 72
Age (donor) [y]	
Mean (SD)	58 (17)
Median (Q1, Q3)	60 (47, 72)
Min, Max	1, 90
Gender (recipient)	
0	234 (30%)
1	545 (70%)
Gender (donor)	
0	359 (46%)
1	420 (54%)
BMI (recipient)	
Mean (SD)	26.5 (4.8)
Median (Q1, Q3)	25.9 (23.2, 29.4)
Min, Max	15.2, 43.2
BMI (donor)	
Mean (SD)	26.2 (4.3)
Median (Q1, Q3)	25.7 (23.5, 27.8)
Min, Max	11.0, 58.8
Weight ratio recipient/donor	
Mean (SD)	1.06 (0.26)
Median (Q1, Q3)	1.02 (0.92, 1.14)
Min, Max	0.57, 4.08
Cold ischemia time [h]	
Mean (SD)	8.6 (3.1)
Median (Q1, Q3)	9.3 (7.5, 10.8)
Min, Max	1.0, 13.0
Abdominal surgery	
0	504 (65%)
1	275 (35%)
Retransplantation	
0	712 (91%)
1	67 (8.6%)
Preoperative hemoglobin [g/dl]	
Mean (SD)	10.87 (2.60)
Median (Q1, Q3)	10.80 (8.70, 12.90)
Min, Max	2.70, 29.00
Platelets	
Mean (SD)	119 (91)
Median (Q1, Q3)	95 (58, 151)
Min, Max	11, 683
International normalized ratio	
Mean (SD)	1.51 (0.81)

Median (Q1, Q3)	1.26 (1.10, 1.60)
Min, Max	0.85, 7.81
Partial thromboplastin time [h]	
Mean (SD)	35 (14)
Median (Q1, Q3)	31 (27, 37)
Min, Max	19, 120
MELD score	
Mean (SD)	19 (10)
Median (Q1, Q3)	16 (11, 28)
Min, Max	6, 40
High urgency	
0	736 (94%)
1	43 (5.5%)
Child-Pugh score	
A	221 (28%)
B	214 (27%)
C	344 (44%)

[1] n (%)

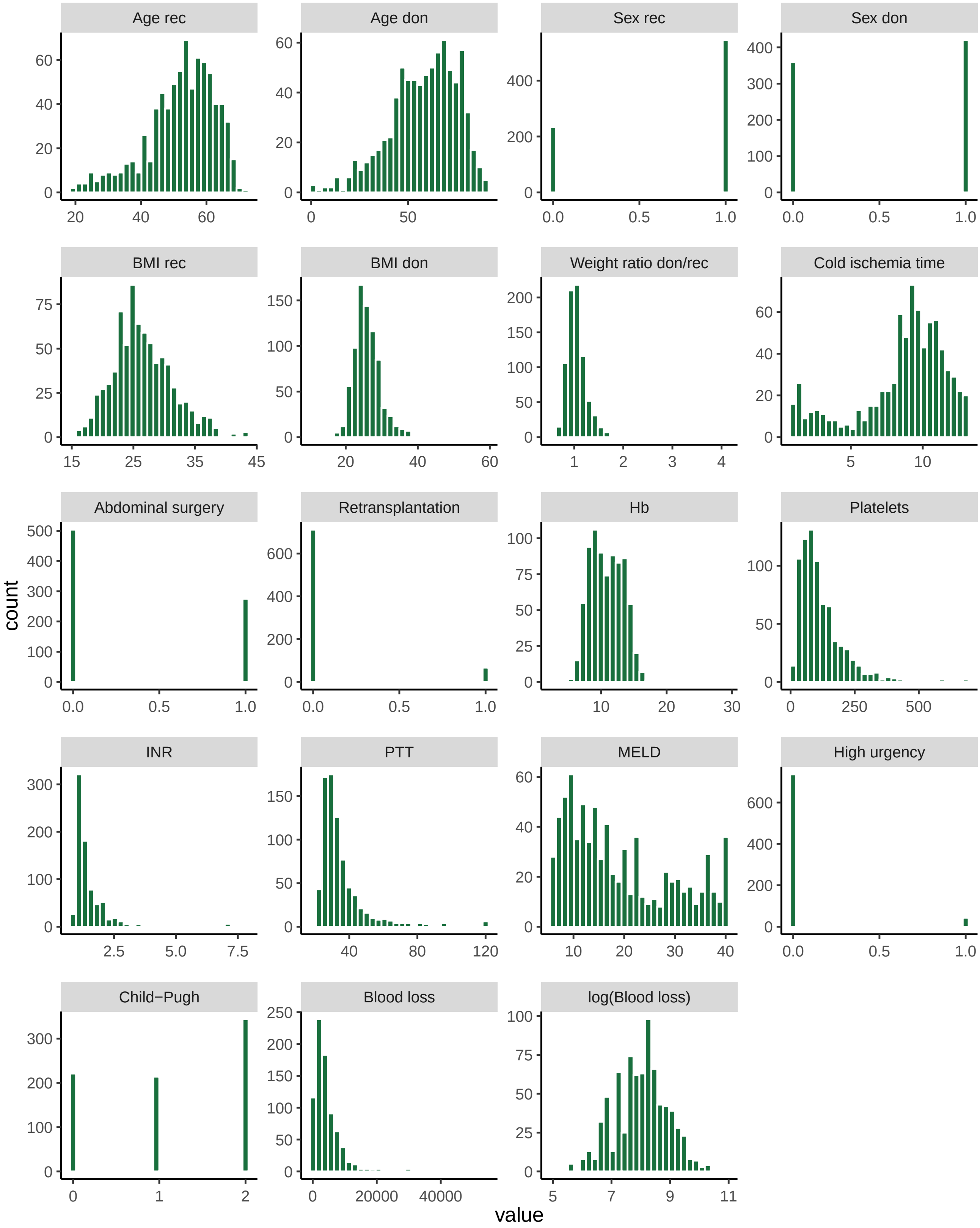

Figure S29: Distribution of the variables of the real data example.

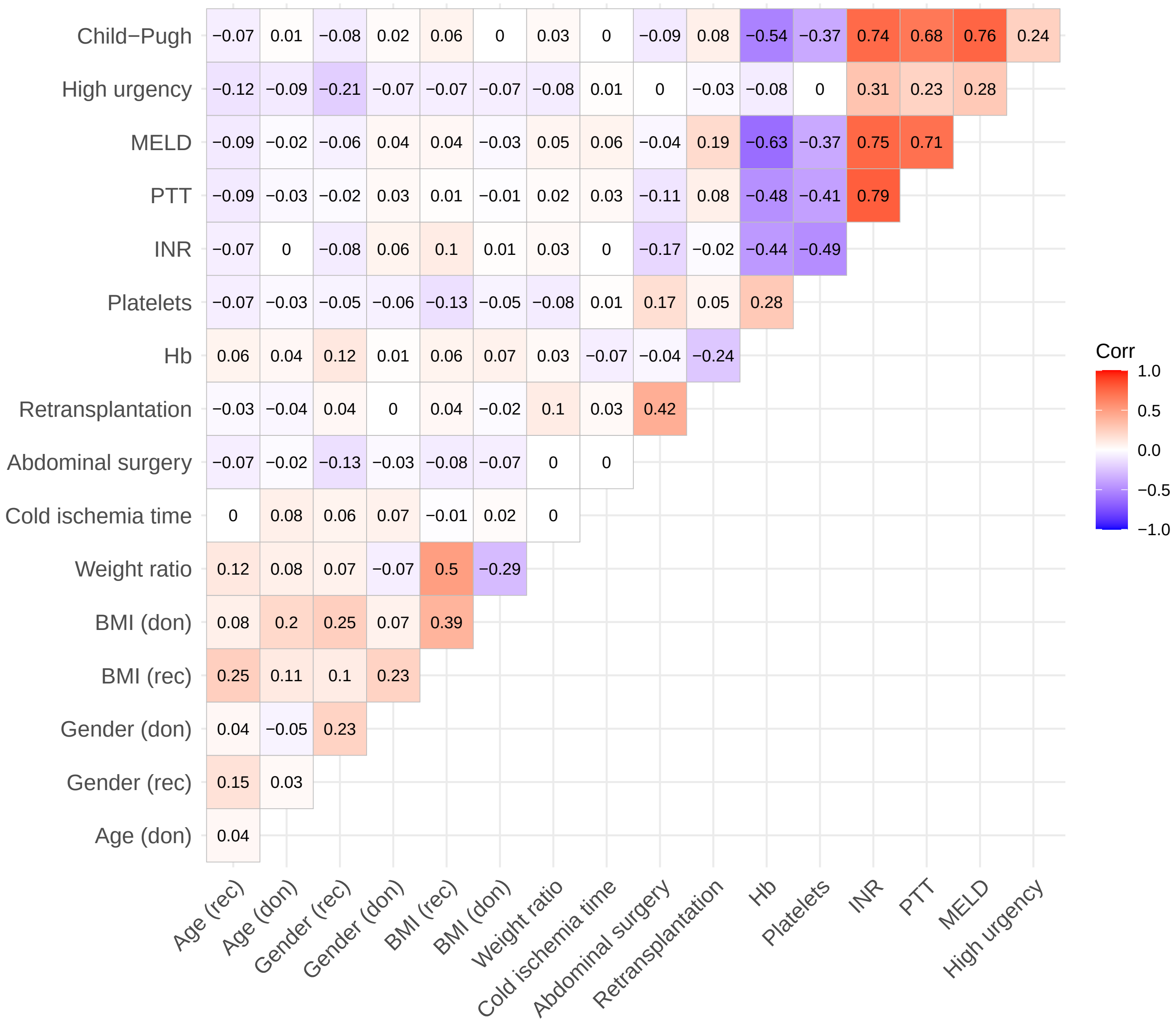

Figure S30: Spearman correlation between the variables of the real data example.
